# Novel Concepts for Inducing Final Oocyte Maturation in *In Vitro* Fertilization Treatment

**DOI:** 10.1210/er.2017-00236

**Published:** 2018-07-02

**Authors:** Ali Abbara, Sophie A Clarke, Waljit S Dhillo

**Affiliations:** Department of Investigative Medicine, Imperial College London, Hammersmith Hospital, London, United Kingdom

## Abstract

Infertility affects one in six of the population and increasingly couples require treatment with assisted reproductive techniques. *In vitro* fertilization (IVF) treatment is most commonly conducted using exogenous FSH to induce follicular growth and human chorionic gonadotropin (hCG) to induce final oocyte maturation. However, hCG may cause the potentially life-threatening iatrogenic complication “ovarian hyperstimulation syndrome” (OHSS), which can cause considerable morbidity and, rarely, even mortality in otherwise healthy women. The use of GnRH agonists (GnRHas) has been pioneered during the last two decades to provide a safer option to induce final oocyte maturation. More recently, the neuropeptide kisspeptin, a hypothalamic regulator of GnRH release, has been investigated as a novel inductor of oocyte maturation. The hormonal stimulus used to induce oocyte maturation has a major impact on the success (retrieval of oocytes and chance of implantation) and safety (risk of OHSS) of IVF treatment. This review aims to appraise experimental and clinical data of hormonal approaches used to induce final oocyte maturation by hCG, GnRHa, both GnRHa and hCG administered in combination, recombinant LH, or kisspeptin. We also examine evidence for the timing of administration of the inductor of final oocyte maturation in relationship to parameters of follicular growth and the subsequent interval to oocyte retrieval. In summary, we review data on the efficacy and safety of the major hormonal approaches used to induce final oocyte maturation in clinical practice, as well as some novel approaches that may offer fresh alternatives in future.

**Essential Points**

- IVF (*in vitro* fertilization) therapy utilizes supraphysiological treatments to simulate many of the physiological processes occurring in the natural human menstrual cycle
- Oocyte maturation is a critical process to the success of IVF treatment, during which the oocyte gains competence for fertilization
- Oocyte maturation is initiated by LH-like exposure that can be provided by human chorionic gonadotropin (hCG), GnRH agonist, recombinant LH, or kisspeptin
- The mode by which oocyte maturation is induced has significant impact on the efficacy of oocyte retrieval, the chance of pregnancy, and the safety of IVF treatment
- Oocyte maturation is part of a continuum with ovulation, and the size of follicles at time of administration and the interval to oocyte retrieval can impact the efficacy of agents of oocyte maturation
- The risk of ovarian hyperstimulation syndrome, a potentially life-threatening complication of IVF treatment that can affect otherwise healthy women undergoing fertility treatment, is strongly related to the agent used to induce oocyte maturationUse of alternative agents of oocyte maturation to hCG can significantly improve the safety of IVF treatment

Infertility affects one in six couples and is recognized by the World Health Organization (WHO) as the fifth most serious global disability (1). This may appear a controversial statement in an overpopulated world; however, Mahmoud Fathalla, former director of the WHO Human Reproductive Program (HRP), explained the rationale for this: “If public health policies encourage couples to delay and plan pregnancies, [then it is] equally important that they are assisted in their attempts to conceive in the more limited time available” (2). The number of *in vitro* fertilization (IVF) cycles carried out across the world is increasing each year, with 1.6% of all children born in the United States in 2015 being conceived through assisted reproductive technology (3).

IVF treatment is a supraphysiological process that simulates many of the physiological processes occurring during the normal human menstrual cycle, namely follicular development, oocyte maturation/ovulation, fertilization, and implantation. During IVF treatment, a pharmacological dose of FSH is used to induce the growth of multiple of ovarian follicles. As follicles grow, an LH surge that could lead to premature ovulation is prevented either through the use of a GnRH antagonist (4, 5), or continuous administration of a GnRH agonist (GnRHa) to downregulate the GnRH receptor (6). Once follicles reach the requisite size, LH exposure is provided to simulate the mid-cycle LH surge, which induces the processes of oocyte maturation and subsequent ovulation (7). Oocyte retrieval is thus precisely timed following provision of LH exposure to retrieve oocytes following oocyte maturation, but prior to the occurrence of ovulation. LH exposure initiates the resumption of meiosis and the maturation of the oocyte from the immature “metaphase I” stage to the mature “metaphase II” stage of development (8). During this process of oocyte maturation, the first polar body is extruded such that a diploid cell transitions toward a haploid gamete and attains competence for fertilization by a spermatozoon (8). Following LH-like exposure, the remainder of the follicle forms the corpus luteum, which produces sex steroids, particularly progesterone, to prepare the endometrium for implantation of the embryo (9). When LH-like exposure is excessive in duration, there is an increased chance of development of a dangerous complication of IVF treatment termed “ovarian hyperstimulation syndrome” (OHSS) (10). OHSS is a predominantly iatrogenic condition that may result in serious adverse consequences for otherwise healthy women undergoing fertility treatment (11).

Thus, the LH-like exposure required to initiate the process of oocyte maturation is a critical step in the success of IVF protocols enabling the efficacious retrieval of mature oocytes, as well as affecting the chance of pregnancy and the safety of IVF treatment. In current IVF protocols, LH-like exposure is provided through either the use of human chorionic gonadotropin (hCG) or GnRHa, which are colloquially referred to as the “trigger” of oocyte maturation (4). hCG has sufficient homology to LH to be able to activate the LH receptor and was the primary and remains the most commonly used trigger of oocyte maturation (4). GnRHa induces endogenous gonadotropin (LH and FSH) release from the pituitary gland and is a safer option, particularly in women at high risk of OHSS (12). Unfortunately, owing to the induction of a shorter duration of LH exposure, the luteal phase is more dysfunctional following GnRHa than hCG, and thus in recent years, there has been an interest in combining the better safety profile of GnRHa with a small dose of hCG to improve pregnancy rates, in so-called “double” or “dual” trigger protocols (13, 14). Although not in common clinical use, recombinant LH (rLH) has also been trialed as a possible alternative to hCG for inducing oocyte maturation (15). More recently, kisspeptin, a neuropeptide that stimulates endogenous GnRH release, has been used to safely mature oocytes during IVF treatment even in women at high risk of OHSS (16–18) (see Fig. 1 for diagram illustrating site of action of each agent used to induce oocyte maturation).

**Figure 1.**
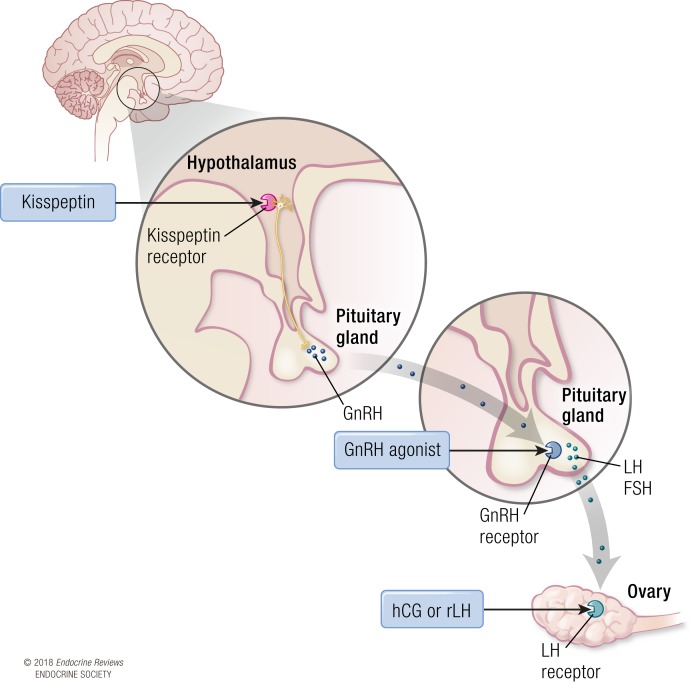
Site of action of inductors of oocyte maturation during IVF treatment. hCG and rLH act directly at LH receptors in the ovary. GnRHas act at GnRH receptors in the pituitary gland to stimulate the secretion of endogenous LH and FSH. Kisspeptin acts at the hypothalamus to stimulate kisspeptin receptors on GnRH neurons and the release of an endogenous pool of GnRH.

Two main stimulation protocols are used to grow follicles and provide the context in which a trigger is administered, namely either the “long” or GnRHa cotreated protocol, and the “short” or GnRH antagonist cotreated protocol. In the short protocol, the GnRH antagonist used is a competitive antagonist, and thus its inhibitory effect can be overcome by GnRH agonism. Thus, the short protocol allows for the use of GnRHa or kisspeptin to induce final oocyte maturation, whereas hCG or rLH can be used in either short or long protocols. The short protocol therefore enables greater flexibility for the hormone stimulus to induce oocyte maturation [see Table 1 (1, 5, 16–34) for summary of agents used to induce oocyte maturation].

**Table 1.** Summary of Agents That Have Been Investigated to Induce Final Oocyte Maturation

Hormonal Stimulus of Final Oocyte Maturation	IVF Protocol*^a^*	Examples	Doses Administered	Site of Action	*t*_1/2_	Time to Peak Concentration of hCG or LH
hCG	Either long or short	uhCG	5000–10,000 IU	Ovarian LH receptors	33.5 h (19, 20)	~20 h (20–22)
		rhCG	250–500 μg		28–29 h (1, 5)	
GnRHa	Short only	Buserelin	0.5–4 mg (23, 24)	Pituitary GnRH receptors	1.3 h (25)	∼4 h (26)
		Triptorelin	0.2–0.4 mg (26–28)		4 h (26)	
		Leuprolide	0.5–4 mg (29–31)		1.5 h (25)	
rLH	Either long or short	rLH	5000–30,000 IU (15)	Ovarian LH receptors	1 h (32, 33)	∼5 h (32, 33)
Kisspeptin	Short only	Kisspeptin-54	Single bolus of 1.6–12.8 nmol/kg or two boluses of 9.6 nmol/kg 10 h apart (16–18)	Kisspeptin receptors on hypothalamic GnRH neurons	28 min (34)	4–6 h (16–18)

Parenthetical numbers are references representing the studies being reported.

^a^ The long protocol indicates a GnRHa-cotreated stimulation protocol, whereas the short protocol indicates a GnRHa-cotreated protocol.

This may be of particular value if the risk of OHSS only becomes apparent during follicular development. Although for some years there were concerns that pregnancy rates could be reduced by using the short protocol (35, 36), several meta-analyses and a large randomized controlled trial (RCT) have established equivalence of these two protocols (37–41). In the United States, there has been an increased use of the short protocol (rising from 35.2% in 2009 to 75.1% in 2015; *P* < 0.0001), to enable more frequent use of GnRHa for oocyte maturation and mitigate the risk of OHSS (42).

In this review, we aim to discuss the predominant hormonal stimuli used to induce final oocyte maturation, with a particular focus on the endocrine requirements for efficacy (retrieval of mature oocytes) and the impact on the luteal phase and safety (risk of OHSS) following the use of either hCG, GnRHa, both hCG and GnRHa in combination, rLH, or kisspeptin.

## Methods

This review was undertaken using a comprehensive literature search of all available articles on PubMed from inception until 31 December 2017 utilizing the search terms “oocyte maturation,” “trigger,” “human chorionic gonadotropin; hCG,” “gonadotropin releasing hormone agonist; GnRH agonist,” “luteinizing hormone releasing hormone; LHRH,” “recombinant luteinizing hormone,” “luteal phase support,” “*in vitro* maturation; IVM,” and “kisspeptin.” Relevant articles commenting on endocrine requirements for oocyte maturation were included in the review. Evidence from randomized clinical trials or meta-analyses was prioritized over retrospective studies where available.

## The Endogenous Menstrual Cycle

Many of the processes in IVF protocols simulate the physiological processes occurring during the natural menstrual cycle, albeit in a supraphysiological manner. During the early follicular phase of the natural cycle, serum estradiol and progesterone levels are low and inhibin B is secreted by small antral follicles (43). Thus, the early follicular phase is characterized by both reduced negative feedback from low sex steroid levels but increased negative feedback on FSH secretion by inhibin B levels (44, 45), overall resulting in an ~30% increase in serum FSH (46–48). This modest rise in FSH stimulates folliculogenesis and aromatase action to increase estradiol production by ovarian granulosa cells (49). In IVF protocols, the modest threshold FSH level for monofollicular development is exceeded by a pharmacological dose of FSH for a duration sufficient to prevent atresia of nondominant follicles and thus induce multifollicular growth (50). As estradiol levels continue to rise, there is a critical switch from negative to positive feedback on GnRH secretion (51), which increases LH synthesis and lowers the GnRH concentration required for LH production (52).

Kisspeptin is a hypothalamic neuropeptide that results in gonadotropin release in both men and women and is requisite for ovulation in women (34, 53, 54). The sensitivity to kisspeptin increases during the preovulatory phase when estradiol levels are highest (53). Although estradiol is key in initiation of the mid-cycle LH surge (55, 56), levels of progesterone during the follicular phase are also influential. Administration of progesterone can advance the timing of the LH surge (55, 57); coadministration of progesterone with estradiol results in an LH surge of greater duration and amplitude than by estradiol alone (58). However, multifollicular development during IVF treatment may alter the hormonal milieu from the natural cycle beyond differences in sex steroid levels alone. For example, gonadotropin surge-attenuating factor (GnSAF) is a molecule produced by ovarian follicles (59) that reduces pituitary sensitivity to GnRH (60) and may act to attenuate the amplitude of the LH surge (61). Differences in GnSAF have been proposed to contribute to the differential sensitivity to GnRH antagonism observed between cycles with monofollicular and multifollicular growth, whereby hypersecretion of GnSAF in cycles with multifollicular development may reduce the degree of GnRH antagonism required to prevent a premature LH surge (61).

## Oocyte Maturation

Final oocyte maturation is the process by which the oocyte resumes meiosis to transition from the metaphase I to the metaphase II stage of development, at which stage it attains competence for fertilization by a spermatozoon (8). The definition can be extended to include the capacity to support embryo development to the blastocyst stage and to live birth (62). It is initiated by LH-like exposure that induces a fall in intraoocyte cAMP and is commonly assessed by the production of a polar body to signify a mature/metaphase II oocyte (63).

In humans, meiosis is initiated during embryogenesis (64), but it is halted at prophase with the nucleus contained within an intact envelope and possessing condensed chromatin (64). At this stage, the oocyte is surrounded by precursors to follicular somatic cells in a single squamous layer, forming the primordial follicle. Oocyte meiotic development remains arrested at this stage until antrum formation (65). Pituitary release of gonadotropins following acquisition of reproductive maturity at puberty stimulates follicular and oocyte growth, resulting in the formation of primary and secondary follicles. Thus, whereas primordial follicle growth is a gonadotropin-independent continuous process (66), secondary recruitment is gonadotropin-dependent. Crosstalk with cumulus cells play an important role in oocyte maturation, providing the oocyte with metabolic support and regulatory cues (67).

### Nuclear maturation

**Figure 2.**
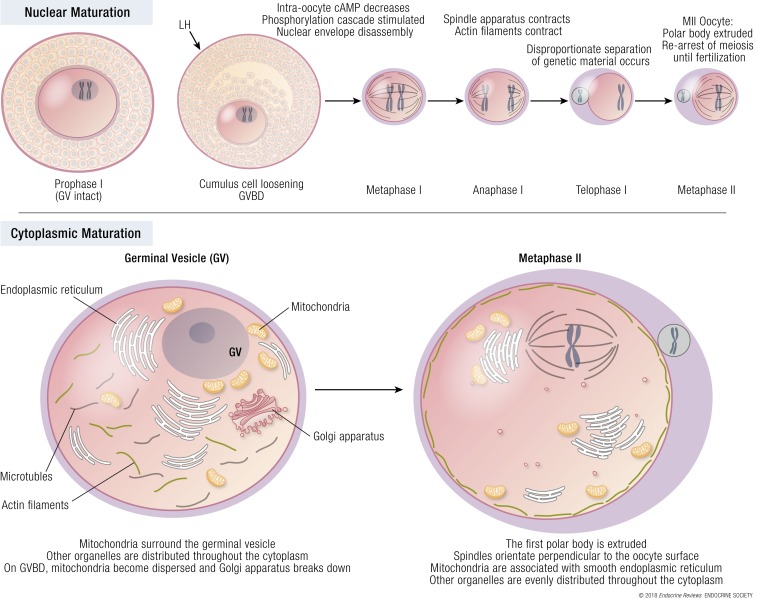
Final oocyte maturation. The midcycle gonadotropin surge causes a decrease in intraoocyte cAMP. The oocyte is removed from meiotic arrest and undergoes a series of coordinated changes affecting both the nucleus and cytoplasm. During nuclear maturation, the haploid metaphase I oocyte extrudes half of its genetic material in a polar body and transitions toward a haploid metaphase II gamete. To achieve this, the germinal vesicle breaks down (GVBD) and chromosomes align along the spindle before separation of genetic material occurs and the polar body is extruded. During oocyte maturation, cytoplasmic and nuclear maturation both occur in related but independent processes. Cytoplasmic maturation prepares the oocyte to meet the metabolic demands of fertilization and embryo growth through changes in organelles. Prior to germinal vesicle breakdown, mitochondria surround the germinal vesicle (GV), and the Golgi apparatus remains intact. By the end of oocyte maturation, mitochondria are associated with smooth endoplasmic reticulum, the Golgi body has been fragmented, and the polar body is extruded. Adapted from Mao *et al.* 2014 (69).

Although nuclear and cytoplasmic maturation are linked processes, cytoplasmic maturation can occur independently of full nuclear maturation (68) [see Fig. 2 (69) for exposition of nuclear and cytoplasmic oocyte maturation]. During the initial growth phase of the oocyte, nuclear chromatin decondenses and is transcriptionally active. As folliculogenesis progresses, the oocyte acquires meiotic competence, as identified by the condensing and nuclear association of chromatin, and the formation of microtubule organizing centers, necessary for spindle formation (70, 71). Yet, although the oocyte now possesses the ability to progress through meiosis, this only occurs if the oocyte is removed from the follicle, with follicular signals ensuring that oocyte development is arrested at prophase I (62). This allows the oocyte to undergo further differentiation between the late antral and periovulatory follicular stages, affording the oocyte developmental competence to sustain embryo development (62). Developmental competence requires a series of nuclear and cytoplasmic cellular events that take place alongside meiotic stages to enable fertilization, DNA replication, and zygote ploidy. The resumption of meiosis is signaled by germinal vesicle breakdown (GVBD). Oocytes then progress through metaphase I in which paired homologous chromosomes align in the middle of the forming meiotic spindle. Nuclear chromosomes then separate, with half the genetic material being extruded in the first polar body, resulting in the formation of a mature, haploid, metaphase II oocyte, with competence for fertilization (64). Normal meiotic spindle morphology in metaphase II oocytes assessed by polarized light microscopy was more likely to result in an euploid embryo (72). A meta-analysis of 10 studies determined that when the meiotic spindle was present, fertilization rates were significantly higher (*P* < 0.0001), as were cleavage rates (*P* < 0.0001) and the proportion of top-quality cleavage embryos (*P* = 0.003) (73). The interval to GVBD is difficult to assess but is estimated to occur at a median of 6.5 hours and the interval between LH receptor activation and the first stage of meiosis is thought to be ~18 hours (74). In humans, spindle assembly typically occurs ~10 hours following GVBD, and ~14 to 20 hours are required between GVBD and polar body extrusion (62). The total duration of nuclear maturation including the time to GVBD has been estimated to be ~20 to 22 hours (75). The oocyte is then arrested at metaphase II until fertilization (68).

### Cytoplasmic maturation

Cytoplasmic maturation prepares the oocyte for nuclear maturation with specific chromatin configurations indicating the likelihood of the oocyte to resume meiosis (67) (see Fig. 2 for diagram of nuclear and cytoplasmic oocyte maturation). Thus, nuclear maturation mainly comprises chromosomal segregation, whereas cytoplasmic maturation involves organelle redistribution, changes in cytoskeletal dynamics, Golgi apparatus, calcium releasing activity, storage of mRNAs, proteins and transcription factors (69). Cytoskeletal changes in microtubules, actin filaments, and chromatin create cell asymmetry and enable polar body extrusion with minimal loss of cytoplasm (69). Although nuclear maturation is apparent by the presence of the extruded first polar body, it is more challenging to assess cytoplasmic maturity in clinical practice (76). Whereas the oocyte only provides half of the genetic material, it provides nearly all membranous and cytoplasmic determinants required for embryogenesis. At metaphase II, the endoplasmic reticulum is redistributed from a fine network into clusters throughout the oocyte, and more functionally competent mitochondria are found beneath the oolemma.

### Maintenance of meiotic arrest

In mammals, prophase arrest is in part maintained by the oocyte itself (64). Cyclin-dependent kinase 1 (CDK1) is a protein expressed by the oocyte that triggers chromosomal condensation and nuclear laminar breakdown, and thus is necessary for the progression from prophase to metaphase I. As the oocyte increases in size, so too does expression of CDK1; however, despite this, the oocyte remains arrested in prophase until it is removed from the follicle. This suggests that inhibitory factors from granulosa cells play an important role in preventing the resumption of meiosis (77). In follicle-enclosed oocytes, LH results in a decrease in cAMP (78) and cyclic guanosine monophosphate (cGMP) to mediate the resumption of meiosis (79, 80). cAMP maintains CDK1 kinase in its inactive form through the action of protein kinase A. Thus, a fall in cAMP allows the formation of active forms of CDK1 kinase to initiate a cascade of events culminating in the resumption of meiosis (64). cGMP is produced by granulosa cells and subsequently diffuses into the oocyte via gap junctions (64), where it competitively inhibits the action of cAMP phosphodiesterase (80). By preventing cAMP hydrolysis, cGMP therefore helps to maintain meiotic arrest.

In summary, human oocytes are arrested at meiotic prophase I until the mid-cycle LH surge signals a series of intracellular changes, including changes in oocyte and granulosa cell cAMP/cGMP levels that result in the resumption of meiosis, and the development of a metaphase II oocyte.

## Current Modes to Induce Final Oocyte Maturation

### hCG

Both hCG and LH are complex heterodimeric glycoproteins with high cystine content. hCG has structural similarity to LH, sharing the same *α* subunit and 85% of the amino acid structure of the *β* subunit (81). This property affords hCG the ability to stimulate the LH receptor (4) and to induce luteinization of granulosa cells and the resumption of meiosis (82).

Although hCG activates the LH receptor, it does not do so in an identical manner to LH. Roess *et al.* (83) demonstrated differences in the binding of LH and hCG to the LH receptor by rotational diffusion. Receptors bound by hCG were immobile, whereas those bound by LH were rotationally mobile, potentially accounting for differences in receptor activation (83). hCG is 30% carbohydrate by weight and has greater glycosylation than does LH, which may also account for differences in receptor binding (83). Furthermore, intracellular signaling following activation of the LH receptor differs depending on the ligand bound (83). hCG possesses higher affinity for the LH receptor than LH, and it is fivefold more potent in stimulating human granulosa cell cAMP activity than equimolar concentrations of LH (84). However, extracellular signal-related kinase 1/2 and AKT (protein kinase B) activation is greater following LH than hCG (84).

In summary, hCG has a greater effect on cAMP and steroidogenic action than does LH, whereas LH has a greater effect on extracellular signal-related kinase 1/2 and AKT signaling, which are antiapoptotic proliferative signals. This difference in action is hypothesized to relate to their physiological roles in the normal menstrual cycle and in early pregnancy, whereby LH plays a key role in inducing oocyte maturation and ovulation, whereas hCG supports the developing embryo and decidua through stimulating steroidogenesis. The translation of these *in vitro* findings is further complicated by the presence of a complex hormonal milieu *in vivo* that can alter these *in vitro* behaviors (85). In conclusion, although both LH and hCG activate the LH receptor, they are not equivalent with regard to both their receptor binding kinetics and the intracellular signaling that they induce.

#### Formulation of hCG

For decades, the only formulation of hCG was derived from the urine of pregnant women (86). However, urinary hCG (uhCG) may contain significant batch-to-batch inconsistencies, and it has the potential for immunological reactions and impurities (86). The advent of recombinant DNA technology has made it possible for recombinant hCG (rhCG) to be synthesized in Chinese hamster ovary cells without the need for any human resource, thereby limiting the above issues (87, 88). uhCG is usually administered intramuscularly, whereas rhCG can be administered subcutaneously. Equivalence between uhCG and rhCG was demonstrated in a phase III double-blinded, randomized controlled study by Driscoll *et al.* (88). The authors compared subcutaneous administration of 250 μg of rhCG with intramuscular administration of 5000 IU of uhCG in 84 women, and they found no significant differences in the number of oocytes retrieved (rhCG 10.8 vs uhCG 10.3), the number of oocytes retrieved per follicle >10 mm on day of trigger (rhCG 90% vs uhCG 80%), the number of mature oocytes (rhCG nine vs uhCG eight), or the number of cleaved embryos (88). A larger randomized controlled study of 297 patients confirmed equivalence, with a similar number of oocytes retrieved following 10,000 IU of uhCG, 250 μg of rhCG, or 500 μg of rhCG (89). Although the higher dose of 500 μg of rhCG resulted in two more zygotes/cleaved embryos than did the lower dose of rhCG, this came at the expense of an increased rate of OHSS (9% vs 3%) (89). Thus, 250 μg of rhCG was recommended for clinical use, being more convenient to administer than uhCG and causing lower rates of OHSS than the higher dose of rhCG. In 2017, Bagchus *et al.* compared 250 μg (~6500 IU) of rhCG subcutaneously with 10,000 IU of uhCG intramuscularly in Japanese and white women (90). Maximal hCG concentrations occurred between 16 and 32 hours before declining during 11 days following administration (90). Interestingly, the mean exposure and mean maximum concentration (*C*_max_) following rhCG was ~20% lower in Japanese women than in white women (90). In Japanese women, *C*_max_ was higher following uhCG than rhCG (141 vs 126 IU/L) and occurred sooner [time to maximum concentration (*t*_max_) 18 hours vs ~22 hours] (90). The half-life was similar at ~35 hours following both preparations and in both ethnicities (90). However, importantly white women chosen for this study were weight-matched to Japanese women with a mean weight of 52 kg, and thus they were not chosen to exemplify a representative white population with higher body weights.

To investigate the possibility that urinary hCG may contain additional factors, such as epidermal growth factor (EGF), that could negatively influence trophoblast function, Papanikolaou *et al.* (91) randomized 119 women to receive either 250 μg of rhCG or 10,000 IU of uhCG to assess pregnancy rates. Live birth rate per protocol was higher following rhCG compared with uhCG (44.1% vs 25.7%) owing to an increased rate of early miscarriage in the uhCG group (28.0% uhCG vs 3.5% rhCG, *P* = 0.01) (91). The authors hypothesized that rhCG may have beneficial effects on placentation compared with uhCG, or that an embryonic factor could account for the difference (91). However, the superiority of rhCG over uhCG has yet to be demonstrated, and two recent Cochrane reviews have found no difference in the rates of oocyte maturation, pregnancy outcomes, or OHSS (39, 92).

#### Dose of hCG

Animal studies in nonhuman mammals provide valuable insight into the effect of hCG dosage on oocyte maturation. In 1997, Zelinski-Wooten *et al.* (93) investigated the effect of varying concentrations of hCG administration in the female rhesus monkey. Following injection of 100 IU, 300 IU, or 1000 IU of rhCG, or 1000 IU of urinary hCG, peak concentrations of bioactive hCG at 2 hours were dose-dependent and similar following uhCG and rhCG (93). The duration of the hCG surge (at levels >100 ng/mL) was also dose-dependent (0 hours for 100 IU, 24 hours for 300 IU, >48 hours for 1000 IU) (93). Fewer animals yielded fertilized oocytes (5 out of 9 animals) at lower doses (100 and 300 IU of rhCG) compared with 1000 IU of rhCG or uhCG (9 out of 10). Furthermore, peak progesterone levels declined sooner after the lower doses relative to 1000 IU of rhCG and uhCG (93). Thus, lower doses were able to induce oocyte maturation and granulosa cell luteinization, but they were insufficient to ensure optimal cytoplasmic oocyte maturation for fertilization and corpora lutea function (93). Hence, a higher dose of hCG influences both the amplitude of hCG level attained, as well as the duration at which hCG levels are maintained over a threshold value.

The terminal half-life of rhCG in humans is estimated to be 29 ± 4.6 hours (21, 22), compared with a half-life of ~30 minutes for endogenous LH (94). In 1987, 302 patients received uhCG at either 2000 IU (n = 88), 5000 IU (n = 110), or 10,000 IU (n = 104) (95). Significantly fewer successful oocyte retrievals occurred following 2000 IU of hCG (77.3%) when compared with either 5000 IU (95.5%) or 10,000 IU (98.1%, *P* < 0.001), suggesting 5000 IU as the minimum effective dose of uhCG (95). Lin *et al.* (96) randomized 164 patients with a body mass index (BMI) of ~20 kg/m^2^ to either 4000 IU or 6000 IU of uhCG and found no difference in either the number of mature oocytes (4000 IU 13.0 vs 6000 IU 11.8), oocyte maturity rate (4000 IU 82% vs 6000 IU 79%), fertilization rate (4000 IU 75.4% vs 6000 IU 80.7%), calculated mature oocyte yield from follicles >10 mm on day of trigger (4000 IU 82% vs 6000 IU 79%), or rates of moderate to severe OHSS (4000 IU 3.6% vs 6000 IU 4.9%) (96). Follicular fluid hCG correlated with serum hCG levels and was proportional to dose (serum levels: 6000 IU 148.3 vs 4000 IU 99.2 IU/L; follicular hCG level: 95.1 vs 62.1 IU/L) (96). Interestingly, clinical pregnancy rates per transfer were higher following 6000 IU of uhCG (4000 IU 36.5% vs 6000 IU 57.0%; *P* = 0.011) (96). Other retrospective studies have concurred that doses of hCG >3000 to 5000 IU are unlikely to confer further benefit on oocyte maturation, and an increase in pregnancy rates was not confirmed with higher doses of hCG (97, 98). Thus, 3000 IU of uhCG is likely to be sufficient for most patients; however, other factors such as body weight may need to be taken into account for individual patients.

“Kisspeptin could be a promising future option, particularly in the woman at high risk of OHSS.”

The impact of serum hCG levels was assessed in 115 patients who received either 5000 IU, 10,000 IU, or 15,000 IU of hCG intramuscularly based on serum estradiol levels (99). Serum hCG levels measured the day following administration suggested a proportional dose response: 113 mIU/mL following 5000 IU, 229 mIU/mL following 10,000 IU, and 361 mIU/mL following 15,000 IU (99). The oocyte yield based on aggregated data (number of oocytes divided by number of follicles >14 mm) did not increase at doses >5000 IU (165% at 5000 IU, 150% at 10,000 IU, and 144% at 15,000 IU) (99). Lin *et al.* (96) categorized their cohort by BMI and found that the serum hCG level achieved was lower in those with higher BMI values. Serum hCG at oocyte retrieval was 110 IU/L in those with BMI <20kg/m^2^ and 90 IU/L in those with BMI of 20 of 25 kg/m^2^ in patients receiving 4000 IU of uhCG (96). Other studies have also reported reduced circulating hCG levels in patients with higher BMI (99–101). Shah *et al.* (102) undertook a prospective randomized crossover trial to investigate whether route of administration (intramuscularly or subcutaneously) or BMI would affect pharmacokinetic properties of hCG. Twenty-two women received either intramuscular uhCG (10,000 IU) or subcutaneous rhCG (250 μg) during the follicular phase of the menstrual cycle (102). The mean concentration (189 vs 72 mIU/mL), maximum concentration (291 vs 142 mIU/mL), and the area under the curve during the first 12 hours (9586 vs 4152 mIU/mL) were higher in patients receiving intramuscular uhCG than subcutaneous rhCG (102). The mean concentration (34 vs 72 mIU/mL), maximum concentration (89 vs 142 mIU/mL), and the area under the curve during the first 12 hours (2352 vs 4152 mIU/mL) were lower in obese women (BMI of 30 to 40 kg/m^2^) than in women with normal BMI (18 to 25 kg/m^2^) (102).

In 2017, Gunnala *et al.* (103) retrospectively reviewed 10,427 IVF/intracytoplasmic sperm injection (ICSI) cycles in which uhCG was administered at varying doses based on serum estradiol on the day of trigger (10,000 IU when estradiol was <1500 pg/mL; 5000 IU when estradiol was 1501 to 2500pg/mL; 4000 IU was estradiol 2501 to 3000pg/mL; and 3300 IU or dual trigger leuprolide 2 mg with 1500 IU of hCG when estradiol was >3000 pg/mL). The number of mature oocytes retrieved, fertilization rate, and number of embryos transferred did not differ by dose of hCG administered (103). However, estradiol levels on the day of trigger correlate with the number of follicles available to provide a mature oocyte, and thus higher doses of hCG could have been administered in patients with fewer follicles and fewer anticipated oocytes had the same dose been used. Oocyte maturation rate (proportion of oocytes retrieved that were mature) varied by serum level of hCG on the morning after administration (68% when hCG was 20 to 30 IU/L, 71% when hCG was 30 to 40 IU/L, 73% when hCG was 40 to 50 IU/L, and 79% when hCG was >50 IU/L; *P* < 0.05) (103). The same group analyzed 18,666 patients with serum *β*hCG levels ≥50 mU/mL and 418 patients with serum *β*hCG levels <50 mU/L on the day following hCG trigger and found that patients with a BMI ≥30 kg/m^2^ had a 21-fold increased risk of having low serum *β*hCG level <50 mU/L (104). Those with a lower *β*hCG level on the day following hCG trigger had lower oocyte maturation rate (76.9% vs 80.5%, *P* < 0.001), lower fertilization rate (62.8% vs 72%; *P* < 0.001), and lower adjusted OR for live birth (adjusted OR = 0.67) (104). Similarly, Matorras *et al.* (105) investigated serum hCG levels at 36 hours following 250 μg of rhCG subcutaneously in 473 women, again demonstrating that serum hCG negatively correlates with BMI (serum hCG = 196 − 3.9 × BMI; *r*^2^ = 0.14) (105). Patients with no oocytes retrieved had a lower serum hCG (77 mIU/mL) compared with those with at least one oocyte retrieved (>116 mIU/mL) (105). The mean number of oocytes retrieved was similar by categories of serum hCG level (8.4 oocytes recovered even in those <50 mIU/mL at 36 hours) and oocyte recovery rates were similar across hCG levels (105). This suggests that although most will have effective oocyte maturation with a standard dose, some individuals with higher BMI could benefit from higher hCG doses to ensure efficacious triggering.

### GnRHa

Although the ability of GnRHas to trigger oocyte maturation has been recognized since the 1970s (106), their potential to induce oocyte maturation was fully realized with the advent of the competitive reversible GnRH antagonists in the 1990s (107). GnRHas displace the GnRH antagonist from the GnRH receptor, leading to receptor activation and gonadotropin release from the pituitary gland (106).

**Figure 3.**
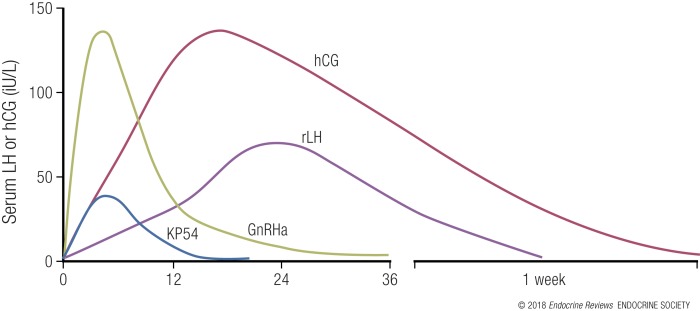
Serum profiles of inductors of oocyte maturation. hCG has a long duration of action with peak serum levels at ~18 hours following administration. GnRHa induces a peak serum LH at ~4 hours following administration (26). Kisspeptin-54 (KP54) also induces a rise in serum LH at 4 to 6 hours following administration but to a lower amplitude than GnRHa (16–18). The profile of rLH is less certain and may peak higher and sooner (15). Serum LH at 24 hours following rLH was 20 IU/L following 5000 IU, 60 IU/L following 15,000 IU, and 90 IU/L following 30,000 IU of rLH (15).

Schally *et al.* (108, 109) first isolated GnRH (at the time more commonly referred to as “luteinizing hormone-releasing hormone”) and synthesized analogs by substituting one or more of the 10 amino acids. Replacement in position 6 or 10 resulted in the formation of analogs that were both more potent than endogenous GnRH and had greater duration of action at the GnRH receptor, with examples including triptorelin, leuprolide, and buserelin (109–111). The half-life of endogenous GnRH is ~2 to 4 minutes; however, the half-life of GnRHa is extended (4, 112) according to amino acid replacement, for example, triptorelin half-life (*t*_1/2_) ~4 hours, nafarelin *t*_1/2_ 3 to 4 hours, leuprolide *t*_1/2_ 1.5 hours, and buserelin *t*_1/2_ 1.3 hours (25). The endogenous LH surge lasts ~48 hours and consists of three distinct phases (113), whereas LH secretion following GnRHa is characterized by two phases, that is, a rapid ascending phase lasting 4 hours, and a longer descending phase (114) (see Fig. 3 for a diagram of hormonal profiles following agents used to induce oocyte maturation). Of relevance, GnRHa activates pituitary GnRH receptors to release both endogenous LH and FSH, whereas hCG possesses only LH-like activity (115). Whereas the mid-cycle FSH surge is not critical for oocyte maturation to occur, FSH is known to increase LH receptor expression in granulosa cells and additionally may directly play a role in the expansion of cumulus–oocyte complexes and oocyte maturation (116–118).

#### GnRHa preparations and dosing regimes

A number of GnRHas have been used to trigger oocyte maturation, with the literature encompassing different agonists and dosages. Several studies have used buserelin at 0.5 mg (23, 24), whereas triptorelin is frequently prescribed at 0.2 mg (27, 28). A recent RCT demonstrated no difference in LH profiles, the number of mature oocytes, fertilization rates, or embryogenesis in oocyte donation cycles following doses of triptorelin between 0.2 mg, 0.3 mg, and 0.4 mg, suggesting that 0.2 mg is at the upper end of the dose-response range (26).

Leuprolide acetate has been used at doses ranging from 0.5 mg (29) to 4 mg (31). In 2015, Pabuccu *et al.* (30) performed an RCT, randomizing 77 women to receive either 1 mg or 2 mg of leuprolide acetate, and they found no significant difference in the number of oocytes retrieved, implantation, or clinical pregnancy rates. Similarly, Parneix *et al.* (119) compared 231 women undergoing ovulation induction with 1 of 13 different regimens for inducing ovulation, including triptorelin, buserelin (both intranasally and subcutaneously), leuprolide, naforelin, or hCG. The authors reported that all regimens resulted in ovulation with no evidence of superiority of one analog over another, and with similar pregnancy rates between groups and the control (hCG) group (119).

In 2017, Şükür *et al.* retrospectively compared patients who received triptorelin at 0.2 mg (n = 63), leuprolide at 1 mg (n = 74), or 10,000 IU of hCG when serum estradiol was <3000 pg/mL (n = 131) (120). The efficacy of oocyte maturation appeared similar between the interventions; the number of mature oocytes divided by the number of follicles >14 mm on the day of oocyte retrieval (calculated on aggregated data) was 120% for triptorelin, 142% for leuprolide, and 128% for hCG (120). Thus, at present, although dosing and type of GnRHa vary in the literature, there is insufficient evidence to support preferential use of any GnRHa over another (4).

#### Efficacy of GnRHas compared with hCG

In 2010, Oktay *et al.* (n = 47) compared leuprolide acetate at 1 mg (n = 27) and hCG at 5000 to 10,000 IU to induce oocyte maturation in women undergoing fertility preservation treatment (121). Although the total number of oocytes was similar between the two groups (GnRHa 16.4, hCG 12.8), a greater number of mature oocytes (GnRHa 11.9, hCG 7.4; *P* < 0.001), higher fertilization rate (GnRHa 84.1%, hCG 74.0%; *P* = 0.027), and more zygotes were observed following GnRHa (121). A prospective randomized controlled study of 122 patients by Humaidan *et al.* (24) reported that although significantly more oocytes were retrieved following 10,000 IU of uhCG (9.7) than following buserelin at 0.5 mg (8.4), the oocyte maturation rate was higher following buserelin (84% vs 68%), leading to slightly more mature oocytes. Nonetheless, luteal levels of progesterone and estradiol were lower in the GnRHa group, corresponding to reduced implantation (GnRHa 3 of 89, hCG 33 of 97) and clinical pregnancy rates (GnRHa 6%, hCG 36%), with increased early pregnancy loss (GnRHa 79%, hCG 4%) (24). In an early meta-analysis, including three studies (122), GnRHa was determined to have similar efficacy to hCG with regard to the number of mature oocytes retrieved, oocyte maturation rate, fertilization rate, and embryo quality.

Thus, GnRHa induces a gonadotropin surge sufficient for oocyte maturation, but it induces a shorter duration of LH exposure than hCG. Whereas this affords an improved safety profile, the same property results in a smaller chance of functional corpora lutea and an increased emphasis on adequate luteal phase support to maintain pregnancy rates.

### rLH

In the mid-1990s, rLH became available as a further therapeutic option for use in IVF treatment. Following intravenous administration, rLH has a similar pharmacokinetic profile to endogenous LH with a distribution half-life of ~1 hour and a terminal half-life of 10 to 12 hours (32, 33, 123). Peak serum LH levels were attained 4 to 5 hours following subcutaneous administration of 10,000 IU of rLH with a terminal half-life of ~21 hours (33). Pierson *et al.* (124) investigated the use of rLH to trigger ovulation during 67 ovulation induction cycles trialing doses of 825, 2750, 5500, 11,000, or 22,000 IU of rLH, or uhCG at 5000 IU. All 50 patients who received doses between 2750 and 11,000 IU ovulated, but 3 of 5 patients in the 825 IU group and 2 of 12 patients in the 22,000 IU group failed to ovulate (124). There was a trend toward a greater rate of ovulation per follicles ≥11 mm with increasing rLH dose (124). Sex steroid levels were increased at days 6 to 9 in a dose-dependent manner (progesterone, 41 to 63.1 nmol/L), but were still higher after 5000 IU of uhCG (progesterone, 97.7 nmol/L) (124). One patient who received 11,000 IU was diagnosed with moderate OHSS (124). The authors concluded that the minimal effective dose of rLH to induce ovulation was 2750 IU.

In 2002, Manau *et al.* (125) randomized 30 women to receive either rLH at 5000 IU subcutaneously or hCG at 5000 IU intramuscularly to trigger oocyte maturation. Patients received additional doses of 5000 IU, 2500 IU, and 2500 IU of hCG or rLH on the days of oocyte retrieval, 2 days later, and 5 days later as luteal phase support (125). A similar number of mature oocytes were retrieved (8.6 with hCG and 7.9 with rLH), with a similar mature oocyte yield (number of mature oocytes from follicles >10 mm) calculated from aggregated data of 58.9% in the hCG group and 60.8% in the rLH group (125). Serum progesterone at 7 days after administration was higher in the hCG group (208 ng/mL) than in the rLH group (148 ng/mL) (125). After ~2.5 embryos were transferred, the implantation rate was similar at 25% to 27.5%; however, 2 of 15 patients in the hCG group developed moderate OHSS compared with none in the rLH group (125). Moreover, hemodynamic changes were less significant following rLH than following hCG, perhaps due to differential intracellular signaling following activation of the LH receptor (125).

In 2001, a multicenter trial across 22 centers in nine countries was conducted to investigate the efficacy of rLH to induce oocyte maturation in comparison with uhCG (15). Two hundred fifty women treated with the long protocol had final oocyte maturation induced by either subcutaneous rLH at doses of 5000 IU (n = 39), 15,000 IU (n = 39), 30,000 IU (n = 26) (15,000 IU plus 10,000 IU administered 3 days after the first injection; n = 25), or intramuscular uhCG at 5000 IU (n = 121) (15). Mean serum LH levels at 24 hours following rLH increased dose-dependently from 23.3 IU/L following 5000 IU to 93 IU/L following 30,000 IU (see Table 2 for summary of clinical trial evaluating rLH for inducing oocyte maturation) (15).

**Table 2.** Summary of Data from Multicenter Trial (15) Using rLH to Induce Oocyte Maturation

Study Design	Population	Dose	Mean Serum LH of hCG at 24 h (IU/L)	Mean No. of Oocytes	Oocyte Maturation Rate (%)	Mean No. of Embryos	Biochemical Pregnancy Rate (%)	Clinical Pregnancy Rate (%)	Live Birth Rate (%)	Any Features of OHSS (%)	Ascites Present (%)	Rates of Moderate OHSS (%)
Human recombinant LH multicenter trial (15):	259 women	5000 IU of uhCG (n = 121)	104	10.8–11.7	78–85	6.3–7.0	31/121 (25)	23/121 (19)	16/121 (13)	83	41–49	12
Prospective randomized, double-blind, dose-finding study with	Age 18–39 y	5000 IU of rLH (n = 39)	23	10.2	86	5.4	6/39 (15)	4/39 (10)	2/39 (5)	51	18	—
GnRHa-cotreated protocol		15,000 IU of rLH (n = 39)	62	11.8	91	6.7	4/39 (10)	3/39 (7)	3/39 (7)	72	21	—
	BMI <32 kg/m^2^	30,000 IU of rLH (n = 26)	93	12.6	57	7.7	6/26 (23)	4/26 (15)	4/26 (15)	77	18	—
		15,000 IU then 10,000 IU 3 d later (n = 25)					8/25 (32)	7/25 (28)	5/25 (20)	80	56	12

The number of oocytes and zygotes following rLH increased dose-dependently by approximately one per dosing category (15). The estimated mature oocyte yield using aggregated data (number of mature oocytes/number of follicles >10 mm) was 70% following 5000 IU of rLH, 77% following 15,000 IU of rLH, and 87% following 30,000 IU of rLH as compared with 68% to 75% following uhCG (15). Two patients had no oocytes retrieved despite comparable serum LH levels (one in the 30,000 IU rLH group and another in the 15,000 IU rLH group). The oocyte maturation rate did not show a clear dose-response and was indeed worse at the highest dose of rLH (15). Despite a mean of 2.4 to 2.8 embryos being transferred, the overall clinical pregnancy rate following a single bolus of rLH was disappointing at 10.6% (11 of 104); however, this was improved in patients who received 15,000 IU of rLH followed by 10,000 IU 3 days later to 28% (7 of 25) to be equivalent to patients who received uhCG at 19% (23 of 121) (15). Concomitant with this, serum progesterone levels in patients who received a single bolus of rLH were significantly lower than the uhCG group, but this was rescued in those who received a further bolus of rLH 10,000 IU 3 days later (243 vs 279 nmol/L) (15). However, the apparent increase in the number of functional corpora lutea as reflected by the increased estradiol and progesterone levels in those receiving a second bolus of rLH came at the expense of an increased incidence of moderate OHSS at 12%, as in the uhCG group (12.4%) (15). The proportion of patients with any features of OHSS showed a dose-response of 51% after 5000 IU of rLH and 80% following 15,000 IU plus 10,000 IU 3 days later of rLH (15). Similarly, there was a relationship between dose of rLH and rise in plasma renin on day 7 after administration (15). Given the half-life and assumed time of peak serum LH levels following rLH, it would be reasonable to speculate that serum LH levels following even the lowest dose of rLH (23.4 IU/L at 24 hours after administration) were likely to have exceeded those found to be effective following GnRHa (~40 IU/L at 12 hours after administration). Although one must be cautious in comparing data across different studies, this study suggested that 5000 IU of rLH was not the top of the dose-response curve for rLH despite achieving such high serum LH levels. A lack of corresponding FSH response may account for some of the reduced efficacy of oocyte maturation in comparison with GnRHa at comparable LH levels. The exact profile of the LH levels achieved and in particular the rise in LH during the first 24 hours was not clear from the data collected, and thus the time to oocyte retrieval may not have been optimal. However, no clear advantage was observed from the use of rLH over hCG and very large doses were required to achieve efficacy. Thus, rLH is not currently in clinical use as an inductor of oocyte maturation. Oral LH agonists are also in development and have been investigated in ovulation induction cycles, but have yet to be evaluated during IVF cycles.

### Kisspeptin

Kisspeptins are a group of hypothalamic arginine-phenylalanine amide peptides encoded for by the KISS1 gene on chromosome 1q32 (126). Kisspeptin isoforms are derived from the proteolytic enzyme cleavage of the 145–amino acid gene product to yield kisspeptins of different amino acid lengths denoted by their suffix (*e.g.*, kisspeptin-54 comprises of 54 amino acids) (126). Their activity at the G protein–coupled kisspeptin receptor is conferred by a common C-terminal decapeptide sequence, equivalent to kisspeptin-10 (127, 128). Kisspeptin acts at the kisspeptin receptor on GnRH neurons in the hypothalamus to elicit endogenous GnRH release, sufficient to induce a subsequent rise in gonadotropin secretion across a range of mammalian species, including humans (129–131) (see Fig. 1 for diagram showing site of action of different inductors of oocyte maturation, including kisspeptin).

The pivotal role of kisspeptin in control of the HPG axis became apparent in 2003 when two seminal papers demonstrated that of loss-of-function mutations affecting kisspeptin signaling resulted in hypogonadotropic hypogonadism (132, 133). Moreover, a girl with an activating mutation in the kisspeptin receptor was reported to have precocious puberty (134). These studies revealed the crucial role kisspeptin plays in regulating the function of the reproductive axis.

Furthermore, evidence from sheep (135) and rodent studies (131) have determined that kisspeptin signaling is necessary for ovulation (54) and that administration of kisspeptin can induce ovulation (135, 136). Kinoshita *et al.* (137) demonstrated that administration of a kisspeptin-neutralizing monoclonal antibody directly into the preoptic area of the hypothalamus of female rats during proestrus was sufficient to prevent ovulation. Matsui *et al.* (136) simulated an IVF protocol in prepubertal rats using pregnant mare serum gonadotropin to induce follicular growth and demonstrated that a subcutaneous bolus of kisspeptin (100 nmol/kg or 6.7 nmol/rat) was able to induce ovulation to the same extent as hCG.

In 2005, Dhillo *et al.* (34) conducted first in humans administration of kisspeptin; a 90-minute intravenous infusion of kisspeptin-54 led to a robust dose-dependent (0.25 pmol/kg/min to 12 pmol/kg/min) rise in serum LH (~fivefold) in healthy men (34). The intravenous half-life of kisspeptin-54 was determined to be 27.6 minutes displaying first-order kinetics (34). Kisspeptin-10, which has a shorter half-life (~4 minutes) than does kisspeptin-54 (~28 minutes), has also been shown to stimulate gonadotropin secretion, both when given as an intravenous bolus (138–140) and when given as a continuous infusion (139, 140).

Kisspeptin also stimulated gonadotropin release in healthy women; however, it was noted that there was variation in response to kisspeptin depending on the phase of the menstrual cycle (53). Although a small subcutaneous dose of kisspeptin-54 (0.4 nmol/kg) elicited a modest mean (±SEM) rise in serum LH from baseline in the follicular phase (0.12 ± 0.17 IU/L), the same dose elicited a much greater rise in the preovulatory phase (20.64 ± 2.91 IU/L; *P* < 0.001) (53). Thus, early rodent studies suggesting that kisspeptin was a key regulator of ovulation (54, 134) and data in women suggesting that kisspeptin could induce an ovulatory LH surge (53) led the group to investigate whether kisspeptin could be used to induce final oocyte maturation during an IVF protocol (18).

#### The use of kisspeptin to induce oocyte maturation

The first trial evaluating the use of kisspeptin-54 to induce oocyte maturation was undertaken in 2014 using an adaptive design (18). Single doses of kisspeptin-54 at 1.6 nmol/kg (n = 2), 3.2 nmol/kg (n = 3), 6.4 nmol/kg (n = 24), and 12.8 nmol/kg (n = 24) were administered 36 hours prior to oocyte retrieval following a standard short protocol (18). Peak plasma levels of kisspeptin were observed at 1 hour following subcutaneous administration resulting in mean serum LH levels of 37.1 IU/L following 6.4 nmol/kg of kisspeptin, and 42.1 IU/L following 12.8 nmol/kg of kisspeptin at 4 to 6 hours following administration with serum LH levels tending toward baseline levels at 12 to 14 hours following administration (18). Of the 53 patients in the study, 51 (96%) had at least one mature oocyte retrieved, and 92% (49 of 53) had at least one embryo available for transfer (see Table 3 for summary of data from three trials using kisspeptin as a novel inductor of oocyte maturation in IVF treatment) (18). The number of mature oocytes increased dose-dependently, although the oocyte maturation rate was similar across doses (18). The mature oocyte yield (percentage of mature oocytes from follicles >14 mm on day of kisspeptin) increased dose-dependently: 36% to 49% at 1.6 to 3.2 nmol/kg, 76% at 6.4 nmol/kg, and 103% at 12.8 nmol/kg (18). Standard luteal phase support was provided by Cyclogest (progesterone) at 400 mg twice daily per vaginal suppository and estradiol valerate 2 mg three times a day orally (18). The live birth rate per protocol at all doses tested was 10 of 53 (19%) and per transfer was 10 of 49 (20.4%) (18).

**Table 3.** Summary of Data From Three Clinical Trials Evaluating Kisspeptin-54 as a Novel Inductor of Oocyte Maturation During IVF Treatment

Study	Study Design	Study Population	Kisspeptin Dosing, nmol/kg	One or More Mature Oocyte, N (%)	Oocyte Yield, % M2 from follicles ≥14 mm	One or More Embryo Formed, N (%)	Embryo Transfer Conducted, N (%)	Biochemical Pregnancy Rate per Transfer, N (%)	Clinical Pregnancy Rate per Transfer, N (%)	Live Birth Rate per Transfer, N (%)	Moderate to Severe OHSS, N (%)
Abbara *et al.* 2015 (16); Abbara *et al.* 2017 (17); Jayasena et al. (2014) (18)	Phase 2, randomized trial with adaptive design	53 women	1.6 (n = 2)	2/2 (100)	49.3	1/2 (50.0)	1/2 (50.0)	1/1 (100)	1/1 (100)		0
	rFSH/ GnRH antagonist cotreatment ICSI protocol	Age 18–34 y	3.2 (n = 3)	3/3 (100)	36.2	3/3 (100)	3/3 (100)	1/3 (33.3)	0/3 (0)	0/3 (0)	
		BMI 18–29 kg/m^2^	6.4 (n = 24)	22/24 (92.0)	76.0	22/22 (100)	22/22 (100)	11/22 (50)	7/22 (32)	10/49 (20.4)	
		Serum AMH	12.8 (n = 24)	24/24 (100)	103.2	23/24 (95.8)	23/24 (95.8)	8/23 (35)	4/23 (17.4)		
		10–40 pmol/L (1.4–5.6 ng/mL)	All doses (n = 53)	51/53 (96.0)	85.1	49/53 (92.5)	49/53 (92.5)	21/49 (42.9)	12/49 (24.5)		
Abbara *et al.* 2015 (16)	Phase 2, randomized trial	60 women	3.2 (n = 5)	4/5 (80.0)	53.0	4/5 (80.0)	4/5 (80.0)	2/4 (50)	1/4 (25.0)	1/4 (25.0)	0
	rFSH/GnRH antagonist cotreatment ICSI protocol	Age 18–34 y	6.4 (n = 20)	20/20 (100)	86.1	18/20 (90.0)	17/20 (85.0)	11/17 (64.7)	10/17 (58.8)	9/17 (52.9)	0
		BMI 18–29 kg/m^2^	9.6 (n = 15)	14/15 (93.3)	85.8	13/15 (86.7)	13/15 (86.7)	11/13 (84.6)	10/13 (76.9)	8/13 (61.5)	0
		Both ovaries intact	12.8 (n = 20)	19/20 (95.0)	121.5	19/20 (95.0)	17/20 (85.0)	8/17 (47.2)	6/17 (35.3)	5/17 (29.4)	0
		High risk of OHSS: AMH ≥40 pmol/L (≥5.6 ng/mL) or AFC ≥23	All doses (n = 60)	57/60 (95.0)	95.1	54/60 (90.0)	51/60 (85)	32/51 (62.7)	27/51 (52.9)	23/51 (45.1)	0
Abbara *et al.* 2017 (17)	Phase 2, randomized, placebo-controlled trial	62 women	Single: 9.6 nmol/kg + placebo 10 h later (n = 31) or	30/31 (96.8)	66.3	30/31 (96.8)	29/31 (93.5)	10/29 (34.4)	7/29 (24.1)	6/29 (20.7)	1 (3.2)
	rFSH/GnRH antagonist cotreatment ICSI protocol	Age 18–34 y	Double: 9.6 nmol/kg + 9.6 nmol/kg 10 h later (n = 31)	31/31 (100)	70.7	31/31 (100.0)	31/31 (100)	16/31 (51.6)	12/31 (38.7)	12/31 (38.7)	0/31 (0)
		BMI 18–29 kg/m^2^	All doses (n = 62)	61/62 (98.4)	68.5	61/62 (98.4)	60/62 (96.8)	26/60 (43.3)	19/60 (31.7)	8/60 (13.3)	1/62 (1.6)
		Both ovaries intact									
		High risk of OHSS: AMH ≥40 pmol/L (≥5.6 ng/mL) 2or AFC ≥23									
All three trials (16–18)			(n=175)	169/175 (96.6)	82.6	164/175 (71.4)	160/175 (91.4)	79/160 (49.4)	58/160 (36.3)	41/160 (26.0)	1/175 (0.57)

Once “proof of concept” that kisspeptin could be used as a trigger of oocyte maturation had been established, a further trial was conducted in 2015 to establish the safety and efficacy of kisspeptin in women at high risk of OHSS (16). Women were identified as being at high risk of OHSS by having a total antral follicle count (AFC) ≥23 or serum anti-Müllerian hormone (AMH) level ≥40 pmol/L to confer an at least fourfold increase risk of OHSS (16). Women were randomized to receive a single subcutaneous bolus of kisspeptin-54 at doses between 3.2 nmol/kg and 12.8 nmol/kg (16). All women were routinely screened for the development of both early OHSS (assessed 3 to 5 days following oocyte retrieval) and late OHSS (assessed 11 days following embryo transfer) (16).

“Shorter durations of the LH surge are sufficient for oocyte maturation.”

The number of mature oocytes again increased dose-dependently (see Table 3) (16). The mature oocyte yield (proportion of mature oocytes from follicles ≥14 mm on the final ultrasound scan prior to kisspeptin-54) was 53% at 3.2 nmol/kg, 86% at 6.4 nmol/kg, 86% at 9.6 nmol/kg, and 121% at 12.8 nmol/kg (16). In this study, luteal phase support comprised of intramuscular progesterone (Gestone at 100 mg daily) in addition to oral estradiol valerate at 2 mg three times a day (16). The live birth rate per transfer was more than doubled in comparison with the first trial at 45% following all doses tested (16). Importantly, although three women (5%) were diagnosed with mild early OHSS, no woman had moderate to severe OHSS (16).

Thus, early results following a single dose of kisspeptin-54 were promising with an overall mean (±SD) oocyte yield of 95% ± 85% and no clinically significant OHSS. A further study was designed to assess the variability in response encountered in some women. This third trial investigated whether prolonging the duration of the LH surge using a second dose of kisspeptin at 10 hours following the first could ensure efficacious oocyte maturation (17). Sixty-two women at high risk of OHSS received kisspeptin-54 at 9.6 mol/kg 36 hours prior to oocyte retrieval (17). Patients were then randomized to receive either saline placebo at 10 hours following the first kisspeptin injection (single group; n = 31), or a second dose of kisspeptin-54 at 9.6 nmol/kg (double group; n = 31) (17). A second dose of kisspeptin improved the proportion of patients achieving an oocyte yield ≥60% from 45% of patients in the single group to 71% of patients in the double group (17). It also eliminated the retrieval of fewer than four oocytes, but importantly not at the expense of an increased rate of ovarian overresponse (17). A unique property of kisspeptin pharmacodynamics became apparent during the trial, whereby a variable rise in LH was observed following the second dose of kisspeptin (17). Those who had a lesser LH response following the first dose had a greater subsequent rise following the second dose of kisspeptin (17). Conversely, patients who already had a robust LH response following the first dose of kisspeptin had minimal further LH secretion following the second dose (17). Thus, the second dose of kisspeptin provided an “individualized” LH response, whereby further LH exposure was only elicited in those patients requiring it (17). This led to the second dose altering the distribution of the number of oocytes retrieved, whereby an increased proportion of patients had an intermediate ovarian response (17). One patient in the single group was diagnosed with moderate early OHSS, as she was admitted for <24 hours for abdominal pain on the day following oocyte retrieval and her symptoms settled with conservative management (17). The live birth rate per protocol was 19% (6 of 31) in the single group and 39% (12 of 31) in the double group (17).

In summary, kisspeptin acts on the hypothalamus to stimulate the release of endogenous GnRH and subsequent gonadotropin release. To date, the trials using kisspeptin suggest that it could be a promising future option particularly in the woman at high risk of OHSS; however, further trials directly comparing kisspeptin to current modes of inducing oocyte maturation are required.

## Endocrine Requirements for Oocyte Maturation and Ovulation

It is relevant to consider evidence from both animal and human studies when evaluating endocrine requirements for triggering final oocyte maturation.

### Data from animal studies

The proportion of the LH surge required for oocyte maturation differs from that required for ovulation and the maintenance of functional corpora lutea. Peluso (141) perfused gonadotropin-stimulated rat ovaries with varying proportions of the gonadotropin surge for 21 hours to assess the minimum gonadotropin exposure required to induce oocyte maturation and ovulation. Whereas only 5% of the gonadotropin surge was required to induce oocyte maturation and maximal progesterone secretion, ovulation only occurred in those exposed to 85% of the gonadotropin surge (141). Similarly, Ishikawa (142) observed that achieving a low level of LH for a longer duration was more able to induce ovulation in proestrus rats than a higher level of LH for a shorter duration. This suggests that a threshold value exists for LH to initiate the process of oocyte maturation/ovulation, and once this level is exceeded, the duration of exposure is more critical for inducing ovulation and supporting functional corpora lutea (142).

The duration of LH exposure required for oocyte maturation and ovulation has been further explored in a series of elegant studies undertaken by the Stouffer group in female macaques. In 1991, Zelinski-Wooten *et al.* (143) compared the following inductors of oocyte maturation in gonadotropin-stimulated female rhesus monkeys: a single intramuscular bolus of 100 IU of hCG, a single subcutaneous bolus of 100 μg of GnRH, three subcutaneous boluses of GnRH at 3-hour intervals, and two boluses of GnRH at 50 μg 8 hours apart. Serum hCG levels remained detectable at 3 days after administration, whereas a single injection of GnRH caused serum LH to peak at 2 hours, and return to baseline by 6 hours (143). Three hourly GnRH injections elevated bioactive serum LH for 8 hours, whereas 8 hourly GnRH injections elevated bioactive serum LH for 14 hours (peak serum LH levels were similar between the groups) (143). hCG induced a greater proportion of oocytes to be in metaphase I or II (86%) than in the GnRH groups (0% to 43%), and only hCG induced a functional luteal phase with progesterone elevation for 11.8 days (143). Thus, 14 hours of LH exposure was found to be insufficient for ovulation. A similar protocol with the following inductors of ovulation were then administered intramuscularly: (1) 1000 IU of hCG, (2) 2542 IU of highly purified urinary human LH, (3) 2542 IU of human LH followed by three injections of 200 IU of human LH at 8 hourly intervals daily during the luteal phase until menses (144). Serum LH levels following intramuscular human LH peaked between 2 and 6 hours but remained elevated for 18 to 24 hours (144). The luteal phase was shorter after a single injection of urinary human LH (<6 days) when compared with hCG or 8 hourly human LH boluses (144). Collectively, these data suggest that short LH surges of 4 to 14 hours were insufficient to induce oocyte maturation and functional corpora lutea (144). The group then investigated extending the duration of the LH surge through either intramuscular injections of: (1) a single bolus of uhCG at 79 μg, (2) two injections of pituitary human LH at 91 μg, (3) a single bolus of rLH at 21 μg, and (4) two injections of rLH (21 μg) 18 hours apart (145). Oocytes and granulosa cells were collected 27 hours after initial injection (144). Following uhCG injection, serum hCG peaked to 1771 ng/mL at 6 hours and remained elevated for >48 hours. Both pituitary LH and rLH elicited a peak within 2 hours (1673 ng/mL after pituitary LH), resulting in an LH surge of >100 ng/mL for 36 to 48 hours after pituitary LH and 18 to 24 hours after rLH (145). The proportions of oocytes resuming meiosis (68% to 76%) were similar in all groups (145). Peak levels of serum progesterone were achieved at 5 days following uhCG injection and resulted in a functional luteal phase of 12.4 days. Peak levels of progesterone in the luteal phase with two doses of pituitary hLH or rhLH were 18.5 and 8.1 ng/mL, respectively, and approached that of uhCG-treated monkeys (39.5 ng/mL) (145). However, a single dose of LH was insufficient to maintain functional corpora lutea (midluteal serum progesterone of 3.4 ng/mL) (145).

In summary, although shorter durations of the LH surge are sufficient for oocyte maturation, a longer duration of at least 48 hours was required to maintain corpora luteal function in the macaques.

### Empty follicle syndrome

In 1986, Coulam *et al.* (146) described the “empty follicle syndrome” (EFS) in four cases from which no oocytes were retrieved following 10,000 IU of intramuscular hCG to induce oocyte maturation, despite apparently normally growing ovarian follicles. The purpose of the inductor of oocyte maturation is to provide sufficient LH-like exposure to initiate and thus synchronize initiation of the process of oocyte maturation over multiple follicles. This allows most oocytes to be mature and ready for retrieval at a defined time point following the trigger, but prior to ovulation. In the absence of sufficient LH-like exposure from the trigger, insufficient oocyte maturation will result, causing EFS. Immature oocytes are more often surrounded by dense unexpanded cumulus cells and are harder to retrieve than mature oocytes (18). Thus, EFS represents a failure of effective triggering and provides a useful defined measure of the very minimum endocrine requirements requisite for oocyte maturation.

EFS is further subcategorized as either “false EFS” whereby an error in administration or reduced absorption of the trigger of oocyte maturation is responsible (two thirds of cases), or “genuine EFS” in which a hormonal response deemed to be sufficient for oocyte maturation is detected but oocyte maturation still does not occur (147). The prevalence of all EFS is estimated to be 0.045% to 3.4% and of genuine EFS to be 0% to 1.1% (148). Some units will conduct a urine test for hCG (signifying a level of at least 10 to 20 IU/L) or a serum LH level following GnRHa to indicate whether EFS is genuine or false as a result from a problem with administration or absorption (149).

To further complicate the diagnosis of genuine EFS, the threshold values for the hormonal response at which oocyte maturation should have occurred are not clearly delineated. The etiology of genuine EFS is thus less well defined. Baum *et al.* (150) suggested that patient predisposition could be important, as EFS was recurrent in 16% of patients, and the prevalence increased significantly with age (151). Rarely there may be hereditable factors that could account for the occurrence of recurrent genuine EFS (152, 153). Yariz *et al.* (154) reported a missense mutation in the LH receptor of two women with infertility and EFS, who could not be rescued with a further dose of hCG. Revelli *et al.* (149) reported a prevalence of EFS of 2.9% among 2729 cycles, excluding false EFS using an hCG level of >20 IU/L. The prevalence of EFS was similar in 2034 high responders triggered with GnRHa and in 1433 unselected cycles triggered with rhCG (3.1% to 3.5%) (155). Blazquez *et al.* (156) identified 74 (0.59%) cases of EFS from 12,483 oocyte donation cycles triggered with 250 μg of rhCG or GnRHa triptorelin at 0.2 to 0.3 mg, of whom 28% were genuine. Of 13 cycles treated with hCG rescue, 85% subsequently had mature oocytes recovered (156). Most of those with EFS (86%) had also previously undergone successful cycles (156). Hasegawa *et al.* (157) detected EFS in 5.1% of cycles and 9.4% of patients, as EFS was recurrent in four patients (12% of women with EFS). These data suggest that whereas EFS can be recurrent in a minority of patients (9% to 16%), cycle-specific factors are more important for most cases.

Beck-Fruchter *et al.* (148) presented the case of a 24-year-old woman who suffered recurrent EFS following hCG, but had successful oocyte retrieval following the addition of the GnRHa triptorelin 40 hours prior to oocyte retrieval in combination with hCG at 250 mg 30 hours prior to oocyte retrieval. This case is instructive in suggesting that there may be some patients who may benefit from variation of standard triggering protocols. In 2014, Haas *et al.* (158) trialed this protocol in eight additional women who had previously experienced ineffective oocyte yields following a single bolus of hCG and significantly improved the number of oocytes retrieved.

Several authors have sought to study the endocrine profiles that predict successful oocyte retrieval to inform the endocrine requirements for oocyte maturation. In 2013, Kummer *et al.* (159) analyzed data from 508 women at high risk of OHSS (>13 follicles measuring >11 mm on day of trigger) in whom oocyte maturation was induced by leuprolide at 1 mg 35 hours prior to oocyte retrieval and determined an incidence of EFS of 1.4% (7 of 508). The mean serum LH at 8 to 12 hours was 59.1 IU/L and serum progesterone was 9.1 ng/mL, whereas in cases of EFS, serum LH level was <15 IU/L (159). In patients with oocytes retrieved, the lowest serum LH was 7.65 IU/L and serum progesterone was 0.7 ng/mL (159). Interestingly, BMI was negatively correlated with both LH rise (*r* = −0.26, *P* < 0.001) and posttrigger progesterone (*r* = −0.22, *P* < 0.001) (159). The number of mature oocytes positively correlated with serum progesterone at 8 to 12 hours (*β* = 0.48), serum LH at 8 to 12 hours (*β* = 0.17), peak estradiol (*β* = 0.15), and negatively correlated with age (*β* = −0.14) and BMI (*β* = −0.04) (159). Hence, despite being the effector of oocyte maturation, serum LH was a poorer predictor of EFS than was the resultant serum progesterone rise. Nevertheless, a low serum LH <15 IU/L at 8 to 12 hours after leuprolide increased the risk of EFS.

Chang *et al.* (160) retrospectively analyzed 1878 patients who received 1 to 2 mg leuprolide 36 hours prior to oocyte retrieval and analyzed hormone levels at ~8 to 13 hours. Median serum LH levels were 51.6 mIU/mL [interquartile range (IQR), 34.2 to 76.8] and median serum progesterone levels were 5.2 ng/mL (IQR, 3.7 to 7.0 ng/mL) measured at a median of 11.1 hours (IQR, 9.6 to 12.2 h) following GnRHa (160). BMI was again found to negatively influence LH rise; serum LH was 64.8 mIU/mL in women with BMI <18.5 kg/m^2^, but 37.3 mIU/mL in women with BMI ≥40 kg/m^2^ (160). Similarly, progesterone was 6.6 ng/mL in women with BMI <18.5 kg/m^2^, but only 3.2 mIU/mL in women with BMI ≥40 kg/m^2^ (160). In 12 patients, successful oocyte retrieval was carried out despite serum LH levels of <8 IU/L (range, 1.8 to 7.8 IU/L); however, all of these patients had a rise in their progesterone values of >4 ng/mL (160). Conversely, three patients had no oocytes retrieved despite serum LH values of >30 mIU/mL, although two of these had serum progesterone values <2 ng/mL (160). Patients with a BMI <22 kg/m^2^ were more than twice as likely to have a failed response to GnRHa (160). The failure also increased by baseline LH taken on cycle day 3, being 13.3% in those with serum LH <1 mIU/mL, 3.6% in those with serum LH 1 to 2 mIU/mL, and 1.8% in those with serum LH >2 mIU/mL (160).

Serum LH prior to administration of GnRH correlates with the subsequent rise in LH (161), and thus patients with low baseline serum LH have an increased likelihood of insufficient LH response to GnRHa.

Collectively, these data highlight that patient factors can lead to a variability in response that is important when counseling patients on an individual basis, even if most patients having the same protocol can be expected to have a positive outcome. Meyer *et al.* (162) observed that 5% of patients who received leuprolide at 2 mg had a serum LH value at 8 to 12 hours of <15 IU/L. Patients with a low serum LH (<0.5 IU/L) on the day of GnRHa increased the risk of having a serum LH level <15 IU/L at 8 to 12 hours following GnRHa from 0.2% to 5.2%, and further to 25% when serum LH was <0.1 IU/L (162). Thus, a low serum LH level prior to GnRHa increases the risk of a suboptimal rise in LH following GnRHa administration and consequently of EFS.

From 175 cycles triggered with kisspeptin using a variety of doses across three studies (16–18), three patients (0.017%) had no oocytes retrieved, all of whom had a serum LH <0.9 IU/L at 12 hours following administration (16–18). A further three patients had no mature oocytes retrieved and had serum LH values <14 IU/L at 12 hours following administration (16–18). However, many patients had mature oocytes retrieved following similar LH values at 12 hours, which could reflect a variation in a patient’s LH requirement for effective triggering (16–18). It is noteworthy that oocyte maturation occurred at seemingly lower LH values following kisspeptin than following GnRHa and rLH. In addition to its hypothalamic role, kisspeptin is known to be present in the ovary (163). Ovarian kisspeptin expression changes in a cyclical manner during the menstrual cycle, and although undetectable in immature oocytes, kisspeptin expression is increased at ovulation (163). Kisspeptin enhances *in vitro* maturation (IVM) of ovine oocytes (164) and porcine oocytes (165). Although, one can speculate that kisspeptin could enhance oocyte maturation in combination with gonadotropin exposure, it is not likely to do so in the absence of a gonadotropin rise (16–18).

In summary, an insufficient rise in LH (<15 IU/L) and progesterone (<3 ng/mL) following GnRHa increases the likelihood of EFS. However, there is crossover in the values obtained in patients with genuine EFS and in women having normal oocyte retrievals.

### Endocrine requirements for efficacious oocyte maturation

Although studying the endocrine profiles to prevent EFS provides an indication of the minimal endocrine requirements for oocyte maturation to occur, this in itself represents the very minimum standard required when assessing trigger efficacy. More usually, one would want to assess the endocrine requirements to provide “efficacious triggering.” The number of mature oocytes can provide a valid indication of the efficacy of triggering during an appropriately powered prospective randomized study if an equal number of oocytes are expected in each group. However, the number of mature oocytes that can be expected will heavily depend on individual patient factors aside from the triggering agent studied, especially the number of follicles available to provide a mature oocyte if effective triggering is provided. Another frequently reported measure is the “oocyte maturation rate” (proportion of oocytes that are mature). However, immature oocytes are also more difficult to retrieve, and thus insufficient triggering can lead to fewer oocytes retrieved and thus a reduction in both the denominator as well as the numerator, making this a less reliable measure of trigger efficacy. A recommended approach for quantifying trigger efficacy is to report the “mature oocyte yield,” whereby the number of mature oocytes retrieved is corrected for the number of follicles on the day of trigger, of a size from which one would expect a mature oocyte to be retrieved if effective triggering is provided (*e.g.*, number of mature oocytes divided by the number of follicles of 12 to 19 mm on the day of trigger) (166). The presence of nuclear oocyte maturation is easily assessed by the appearance of a polar body denoting a metaphase II oocyte; however, cytoplasmic oocyte maturation requires more detailed imaging to fully assess, which may not be readily available in many centers. The fertilization rate is used by some authors as a surrogate measure to indicate that cytoplasmic oocyte maturation has occurred.

Oocyte yield was assessed by Chen *et al.* (167) in 91 patients who received GnRHa triptorelin at 0.2 mg 34 to 38 hours prior to oocyte retrieval. Mean serum LH at 12 hours after GnRHa was 46.6 IU/L (range, 9.7 to 151.2 IU/L), but 5.5% of patients had a serum LH <15 IU/L (167). The oocyte yield (number of oocytes as a proportion of follicles >10 mm on day of GnRHa) was 38% and oocyte maturation rate was 77% in patients with a 12 hour serum LH <15 IU/L as compared with an oocyte yield of 69% to 75% and oocyte maturation rates of 79% to 84% in patients with higher LH values (167). Serum LH on the day of GnRHa administration was 0.7 IU/L in patients with a 12 hour serum LH <15 IU/L as compared with 1.6 IU/L in remaining patients, again reinforcing the concept that a low endogenous serum LH may predict an inadequate rise in LH following GnRHa triggering (167). No significant differences were observed in other outcomes, including oocyte maturation, fertilization rate, and clinical pregnancy rate (although cycles were supplemented with hCG for luteal phase support) (167). Similarly, in 2011, Shapiro *et al.* (168) found a modest reduction in oocyte yield (defined as proportion of oocytes from follicles ≥10 mm on day of GnRHa) and mature oocyte yield (defined as ratio of mature oocytes to the number of follicles ≥10 mm on day of GnRHa) when serum LH at 12 hours was <52 IU/L, but a more dramatic reduction when the serum LH was <12 IU/L. Oocyte yield was 70% when 12 hour LH was <12 IU/L, 79% when 12 hour LH was <52 IU/L, and 86% when 12 hour LH was >52 IU/L (168).

### Combination of hCG and GnRHa—a role of FSH in oocyte maturation?

HCG is an effective inductor of oocyte maturation but provides only LH-like exposure, suggesting that the mid-cycle FSH surge observed in the natural cycle is not requisite for successful oocyte maturation. A potential advantage of GnRHa and kisspeptin over hCG and rLH is the concomitant release of FSH in addition to LH-like activity. FSH can promote formation of LH receptors in luteinizing granulosa cells, nuclear maturation, and cumulus expansion (116, 169, 170). In 1998, Zelinski-Wooten *et al.* (171) reported that a large bolus of 2500 IU of recombinant FSH in isolation was able to induce oocyte maturation to a similar extent as hCG in the female rhesus monkey. Peak FSH concentrations were observed 2 to 8 hours following injection and had returned to baseline by 96 hours (171). However, FSH alone was unable to sustain the luteal phase, suggesting that LH action is additionally required for maintaining corpus luteal function (171). Consistent with this, Bianchi *et al.* (172) reported the case of a 36-year-old woman with PCOS who was treated with a long IVF protocol, but administered 2100 IU of recombinant FSH rather than hCG in error. The patient had nine oocytes retrieved, of which eight were mature, and treated oocytes underwent normal fertilization, although pregnancy did not ensue (172). Furthermore, Rosen *et al.* (173) noted that intrafollicular FSH levels corrected for follicular size were higher in follicles that yielded an oocyte. In 2011, Lamb *et al.* (169) conducted a randomized double-blind placebo controlled trial in 188 women to assess whether additional recombinant FSH could enhance oocyte maturation if given concomitantly with hCG at time of trigger. Women treated with the long protocol with serum estradiol levels <4500 pg/mL were randomized to receive either hCG at 10,000 IU with 450 IU of recombinant FSH, or hCG alone (169). Fertilization rate was significantly improved with supplementation of recombinant FSH (63% vs 55%) and a greater likelihood of oocyte recovery was observed, defined as the rate of obtaining an oocyte from a single mature-sized follicle on each ovary (70% vs 57%) (169). Clinical pregnancy rate (56.8% vs 46.2%) and ongoing/live birth rates (51.6% vs 43.0%) were not significantly improved (169).

Owing to the duration of the LH surge following GnRHa being insufficient to support functional corpora lutea and support implantation, there is increasing interest in using a combination of GnRHa with a small dose of hCG. Some investigators have given them simultaneously (termed “dual trigger”), whereas others have administered hCG later to rescue the luteal phase (termed “double trigger”).

*“The need for additional GnRHa during triggering is yet to be clearly demonstrated and further data are required.”*

In 2014, Haas *et al.* (158) conducted a pilot study assessing whether patients with a low oocyte yield (defined as the ratio of the number of oocytes retrieved divided by the number of follicles >14 mm on day of trigger of <50%) in response to hCG could be improved by the addition of a GnRHa and increasing the interval to oocyte retrieval. Eight patients with a low oocyte yield following hCG at 250 μg administered 36 hours prior to oocyte retrieval subsequently received 0.2 mg of triptorelin 40 hours prior and hCG at 250 μg 34 hours prior to oocyte retrieval (158). The number of oocytes retrieved improved from 2.3 to 7, the number of oocytes from follicles >10mm improved from 19% to 80%, and the number of oocytes from follicles >14 mm improved from 24% to 118%, with three of eight patients having ongoing pregnancies (158). The same group showed a similar improvement in 12 patients with low oocyte maturation rates (<66%) (174). Again, improvements were seen in the number of oocytes with double trigger (10.4 vs 8.0), the number of mature oocytes (6.5 vs 3.7), the oocyte maturation rate (70% vs 47%), and the ongoing pregnancy rate (50% vs 0%) (174). Since two interventions were investigated simultaneously, it is difficult to identify which intervention resulted in the improvements observed. Although there is biological plausibility to the addition of GnRHa providing additional LH and FSH exposure, and several other nonrandomized studies have reported that patients with poor oocyte maturation in a first cycle triggered with hCG can have improved outcomes in a subsequent cycle if supplemented with GnRHa (76, 175, 176), such a study design may be susceptible to “regression to the mean” if randomization to a control intervention is not also assessed.

In 2008, Schachter *et al.* (177) conducted an RCT in 221 short protocol IVF cycles to compare hCG at 5000 IU alone (n = 103), with hCG and triptorelin at 0.2 mg given in combination (n = 97) at 34 hours prior to oocyte retrieval. Participants had at least one previous failed IVF protocol, but not with EFS (177). One of 106 patients (0.9%) in the hCG group and 3 of 105 patients (2.9%) in the GnRHa-supplemented group had no oocytes retrieved and a further 2 (1.8%) of the hCG group and 5 (4.8%) of the GnRHa-supplemented group had no fertilization (177). However, in contrast to these results, patients in the hCG group had 7.9 oocytes retrieved and the GnRHa-supplemented group had 9.9 oocytes retrieved (177). Serum FSH on the day of oocyte retrieval was higher in the GnRHa-supplemented group (11.3 vs 6.3 IU/L), as was serum LH (5.2 vs 3.3 IU/L) (177). The ongoing pregnancy rate per embryo transfer was 22% in the hCG group and 36% in the GnRHa-supplemented group (177).

In 2014, Decleer *et al.* (178) conducted an RCT comparing 59 patients who received 5000 IU of hCG alone with 61 patients who concomitantly received GnRHa at 0.2 mg of triptorelin 36 hours prior to oocyte retrieval. A mean of 10.3 mature oocytes were retrieved in the GnRHa-supplemented group as compared with 9.2 mature oocytes in those receiving hCG alone (178). There were 1.4 more zygotes formed in the GnRHa-supplemented group with 74% of patients having a high-quality embryo formed, as compared with 48% of the hCG alone group (178). The ongoing pregnancy rate was 44% in the hCG group and 31% in the GnRHa-supplemented group, with no cases of OHSS reported in either group (178). As expected, serum LH was ~60 IU/L 1 day one after receiving hCG with GnRHa, but interestingly serum FSH rose in both groups from ~8 IU/L on the day of trigger to ~15 IU/L 1 day after the day of hCG, and to 35 IU/L in the GnRHa-supplemented group (178). Serum progesterone was highest on the day of oocyte retrieval in both groups at ~10 to 15 ng/L (178).

Ding *et al.* (179) conducted a meta-analysis including 527 women from four eligible RCTs to investigate the efficacy of the dual trigger in comparison with hCG alone [Schachter *et al.* (177) in 2008, Decleer *et al.* (178) in 2014, Kim *et al.* (180) in 2014, Mahajan *et al.* (181) in 2016]. The analysis did not demonstrate any difference in the number of oocytes, mature oocytes, zygotes, or implantation rate, although it did find an increase in the pregnancy rate in the GnRHa-supplemented group as compared with hCG alone (relative risk, 1.55; 95% CI, 1.17 to 2.06) (179). In summary, additional FSH exposure is suggested to enhance oocyte maturation, although LH/hCG play a dominant role and the additional impact of FSH is likely to be small. Although several reports have suggested that GnRHa supplementation may improve oocyte maturation in patients with a history of poor oocyte maturation, the need for additional GnRHa during triggering is yet to be clearly demonstrated and further data are required. In the past, it had been suggested that GnRHa may directly impair implantation rates perhaps through a direct negative action on the endometrium, or through other effects beyond those due to a shorter duration of action than hCG; however, these data would suggest that such effects are unlikely to be clinically significant in practice.

### Interval between hCG and oocyte retrieval

There is a continuum between the processes of oocyte maturation and ovulation if sufficient LH-like exposure is provided. Thus, it is critically important to schedule oocyte retrieval at a precise interval following administration of the agent of oocyte maturation, such that it takes place following oocyte maturation, but prior to ovulation. If the interval is too long, ovulation may have occurred prior to retrieval and oocytes will no longer be present within the follicles, whereas if the interval is too short, insufficient time may have been provided for optimal oocyte maturation and oocyte retrieval is more difficult.

In the natural cycle, the median interval from the rise in LH to ovulation is 32 hours (95% CI, 24 to 38) and following the peak in LH is 16.5 hours (95% CI, 10 to 23) (182). Ovulation occurs in 90% of women between 16 and 48 hours after the first significant rise in LH and between −3 and 36 hours after the peak (182). Andersen *et al.* (182) conducted a study in clomiphene-stimulated cycles to assess the time of first ovulation following intramuscular hCG. In 66% of cases, the largest follicle was the first to rupture and the mean time to ovulation following hCG was 38.3 hours, although the range was between 34 and 46 hours (183). In 1990, Nader and Berkowitz (184) found that ovulation occurred within 36 hours following intramuscular hCG administration in some women, and thus suggested that an interval <35 hours between hCG administration and oocyte retrieval be used. This was in keeping with evidence from clomiphene-stimulated cycles that suggested that the interval between hCG administration and ovulation was <34 hours for most patients, but could extend to >39 hours in some patients (185). These data highlight the variability in time to ovulation among different women; however, ovulation could be expected to occur sooner after ovulation induction cycles than in cycles with multifollicular development.

Nargund *et al.* (186) studied 533 long protocol cycles with an interval between intramuscular 10,000 IU of uhCG and oocyte retrieval ranging between 33 and 41 hours. No significant differences were observed in the ratio of oocytes retrieved divided by the number of follicles punctured among the interval groups (33 to <36 hours, 63.8%; 36 to <38 hours, 60.9%; 38 to <41 hours, 62.5%) (186). Lengthening the interval up to 41 hours did not increase the proportion of cycles affected by premature ovulation, nor were there significant differences in the number of oocytes retrieved, fertilization rates, or clinical pregnancy rates (186). Similarly, in 2000, Bjercke *et al.* (187) found no difference in number of oocytes retrieved, oocyte–cumulus complex quality, embryo quality, implantation, or pregnancy rates when women were randomized to undergo oocyte retrieval at either 34 hours or 38 hours following hCG. Bosdou *et al.* (188) randomized 156 normo-ovulatory women treated with the short protocol to either an interval of 36 hours or 38 hours following 250 μg of rhCG and oocyte retrieval and found no difference in oocyte retrieval rate (number of cumulus–oocyte complexes divided by number of follicles ≥11 mm on day of hCG), maturation rate (88% vs 80%), fertilization rate (58% vs 50%), or number of zygotes. Importantly, no patient ovulated prematurely due to the extension of interval between hCG and oocyte retrieval (188). Interestingly, a case report has suggested that mature oocytes can be retrieved following a prolonged interval of ~60 hours after hCG administration (189). Successful fertilization and embryo transfer were performed, although no subsequent pregnancy ensued, suggesting that the interval between hCG and oocyte retrieval can be extended from the standard 36 hours in many patients without causing ovulation.

In 2011, Wang *et al.* (190) conduced a meta-analysis of five trials investigating the interval between hCG and oocyte retrieval (186, 187, 191–193), including a total of 895 patients. Oocyte maturation rate was significantly higher when oocyte retrieval was performed >36 hours after hCG administration, compared with <36 hours after administration (risk ratio, 0.67; 95% CI, 0.62 to 0.73) (190). There was no difference observed in fertilization rate, implantation rate, or clinical pregnancy rate (190). However, the studies included were heterogeneous, with timing of the “short interval” of oocyte retrieval ranging from 33 to 36 hours, and the “long interval” ranging from 36 to 41 hours (190).

Ghasemian *et al.* (194) investigated 126 cycles in which oocyte retrieval had been carried out at either 35, 36, 37, or 38 hours following 10,000 IU of uhCG and found that the oocyte maturation rate increased with interval to oocyte retrieval at 35 hours [89.2% (n = 20)], 36 hours [90.5% (n = 45)], 37 hours [92.0% (n = 38)], and 38 hours [94.0% (n = 23)]. Oocyte morphology (extracytoplasmic, referring to the perivitellin space and zona pellucida; cytoplasmic quality, referring to the presence of dark or granular cytoplasm suggestive of aggregates of smooth endoplasmic reticulum, and presence of vacuole) was most frequently normal in those with a 36-hour interval: retrieved oocytes at 35 hours 47.4% (n = 20), at 36 hours 64.1% (n = 45), at 37 hours 52.9% (n = 38), and 38 hours 25.8% (n = 23) (194). Thus, a longer interval to oocyte retrieval may result in fewer good-quality oocytes and fewer high-quality embryos (194), although data supporting the value of oocyte morphology remain conflicting (195).

Thus, overall these data suggest that there is likely to be significant interindividual variation in the interval between the LH surge and the time to ovulation in the natural cycle. Similarly, in stimulated cycles, there is evidence to suggest that extending the interval between hCG administration and oocyte retrieval beyond the standard 36 hours is unlikely to lead to the frequent occurrence of premature ovulation. Data from studies with the double trigger discussed below, in which a GnRHa is administered 40 hours prior to oocyte retrieval in combination with hCG 34 hours prior, also suggest that for some patients there may be a benefit to extending the interval to oocyte retrieval in those not responding well to standard protocols (158). However, to date there is insufficient evidence to suggest an improvement in outcomes with longer intervals between trigger and oocyte retrieval for most patients, although further studies taking into account follicle size profiles on day of trigger and hormonal response following trigger, especially in patients with suboptimal responses to standard protocols, are required to fully resolve this issue.

### Follicle size at time of trigger administration

During controlled ovarian stimulation, follicles are stimulated to grow under the action of a pharmacological dose of FSH. It is widely accepted that follicles that are too small are less likely to yield mature oocytes following LH-like exposure (196). Conversely, ovarian follicles that grow too large can become “postmature” and are also less likely to yield a mature oocyte (197). Thus, most IVF centers will monitor follicular size and administer the trigger of oocyte maturation, once follicles are deemed to have grown to an appropriate size, typically judged as two to three lead follicles >17 to 18 mm in diameter.

*“Some patients who received kisspeptin achieved mature oocyte yields over 100%.”*

Knowledge of the size of follicles at the time of trigger expected to yield an oocyte can also enable the more accurate quantification of trigger efficacy. In 2007, Shapiro *et al.* (198) observed that GnRHa resulted in significantly more oocytes retrieved (28.8) than for hCG (21.6). However, those treated with GnRHa had more follicles on the day of trigger (GnRHa 34.2; hCG 21.7), making it difficult to accurately compare trigger efficacy between the two groups (198). In view of this, Shapiro *et al.* (168) proposed the concept of “oocyte yield,” whereby the number of oocytes is corrected for the number of follicles on the day of trigger administration. They reported a mature oocyte yield defined as proportion of mature oocytes from follicles ≥10 mm on the day of trigger of 63% following GnRHa (168). However, the low percentage encountered following an established dose of an effective trigger suggests that the follicle size denominator chosen empirically may have been too broad, perhaps encompassing follicles that were too small to yield an oocyte. Other denominators have been used, with some authors reporting both the number of follicles ≥14 mm and the number of follicles ≥10 mm on the day of trigger to account for different estimations of oocyte yield (158). Studies evaluating the efficacy of kisspeptin to induce oocyte maturation have used a denominator of follicles ≥14 mm on day of trigger and achieved a reasonable dose response (16–18). Similarly, some patients who received kisspeptin achieved mature oocyte yields over 100%, suggesting that follicles <14 mm may have also contributed to the number of mature oocytes retrieved.

There are limited data to justify the categories of follicle size on day of trigger used to estimate oocyte yield in the current literature, and none of the thresholds includes an upper limit for follicle size at which postmature follicles may become more prevalent. There does, however, exist relevant data on the follicle sizes on the day of oocyte retrieval that are most likely to yield an oocyte.

Rosen *et al.* (199) observed that the odds of retrieving a mature oocyte from a follicle 13 to 15 mm on the day of oocyte retrieval in size is reduced by 70% compared with follicles >18 mm. Wittmaack *et al.* (200) reported that follicles with a volume <1 mL (~12.4 mm) or >7 mL (~23.7 mm) on the day of oocyte retrieval had lower oocyte yields (59%) when compared with those between 1 and 7mL (~74% to 85%). Dubey *et al.* (201) determined that fertilization rates were increased in oocytes from larger follicles on the day of oocyte retrieval (10 to 14 mm, 57.9%; 16 to 22 mm, 69.9%; 22 to 26 mm, 73.9%). Ectors *et al.* (197) found that fertilization rates were greatest in follicles 16 to 23mm in size on the day of oocyte retrieval (68%), compared with either those <16 mm (56%) or those >23 mm (56%). Oocyte maturation rates were >95.3% in those follicles >23 mm on the day of oocyte retrieval, as compared with 75.3% in those <16 mm (197). Overall, data suggest that follicles of 16 to 22 mm on the day of oocyte retrieval are most likely to yield an oocyte (196).

In 2016, Hu *et al.* (202) analyzed 492 IVF cycles treated with the short protocol and categorized patients by the proportion of follicles ≥10 mm on the day of trigger that were also ≥17 mm as low proportion (30% of follicles ≥10 mm were also ≥17 mm), middle proportion (30% to 60% of follicles ≥10 mm were also ≥17 mm), or high proportion (>60% of follicles ≥10 mm were also ≥17 mm). The number of oocytes retrieved was greatest in patients with a low proportion of follicles ≥17 mm (oocyte number: low, 9.2; middle, 7.6; high, 7.2), suggesting that follicles >17 mm on day of trigger contribute less to the number of oocytes retrieved than do smaller follicles (202). Oocyte maturation rate was low (85%), middle (89%), and high (88%) and fertilization rate was low (72%), middle (74%), and high (75%) (202).

There also exist data investigating the impact of adjusting the day of trigger administration, although no clear consensus was apparent. Kolibianakis *et al.* (203) randomized patients to receive either the trigger once three or more follicles had reached ≥17 mm in diameter, or to delay administration of the trigger by 48 hours thereafter. Delayed triggering resulted in 1.3 fewer follicles of 11 to 14 mm and 3.1 more follicles of ≥17 mm with an associated rise in progesterone of 0.4 ng/mL and detrimental effects on pregnancy potential, but a nonsignificant increase of 1.2 oocytes retrieved (203). Similarly, Kyrou *et al.* (204) compared administration of hCG once three follicles were ≥16 mm in diameter (early), or 24 hours later (late), and found that delaying triggering increased the number of mature oocytes retrieved (early 6.1, late 9.2, *P* = 0.009) with an associated rise in serum progesterone levels by 0.3 ng/mL. Mochtar *et al.* (205) randomized women to receive hCG once the lead follicle was either 18 mm or 22 mm, and they observed that those with a lead follicle of 22 mm had a greater number of follicles of 20 to 22 mm on day of trigger (3.95 vs 0.02) and an increase of two oocytes retrieved. Vandekerckhove *et al.* (206) found that a 24-hour delay in trigger administration of patients with three or more follicles of ≥18 mm (and 30% to 50% of follicles ≥10 mm were also ≥18 mm) increased the number of mature oocytes retrieved by 2.4, but only when serum progesterone was ≤1 ng/mL. This could suggest that larger follicles with evidence of luteinization may less likely yield an oocyte than do larger follicles without evidence of luteinization. Conversely, Tan *et al.* randomized patients to receive hCG either once the lead follicle was 18mm with a further 2 follicles >14mm, or 1 day later, or 2 days later, and observed no difference in the number of oocytes retrieved (207). Similarly, Tremellen *et al.* (208) found that patients with “ideal” timing of the hCG trigger (defined as two or more follicles of ≥17mm, with most follicles ≥14 mm) had similar outcomes to patients triggered either a day earlier or later. In 2014, Chen *et al.* (209) conducted a meta-analysis including seven RCTs and 1295 IVF cycles comparing hCG administration as soon as three or more follicles were ≥17 mm in size (“early”) compared with either 24 or 48 hours later (“late”). Fertilization rates were higher in the 48 hours later group (*P* < 0.0001), although this result was predominantly attributable to the results of one study, and overall no significant benefit was observed (209).

In summary, the size of follicles at the time of trigger can influence the likelihood that LH-like exposure can induce oocyte maturation. Most reproductive medicine centers administer hCG once two to three lead follicles are 17 to 18 mm in diameter. When follicles grow as a tight cohort behind the lead follicle, the lead follicle provides a reasonable representation of all of the follicles. However, when follicle sizes on day of hCG are more disparate, the lead follicle may perform less reliably as a representation of all follicles. Data on specific follicle sizes that are most likely to yield an oocyte have predominantly been generated on the day of oocyte retrieval, at which time follicles of 16 to 22 mm are thought to be most likely to yield oocytes (196). Data from our own group suggest that follicles of 12 to 19 mm on the day of trigger contribute most to the number of mature oocytes retrieved (166). Indeed, patients with a greater proportion of their follicles within this range had more mature oocytes retrieved (166). These data also allow for a data-driven estimation of trigger efficacy. Thus, we recommend that “mature oocyte yield” defined as the proportion of mature oocytes retrieved from follicles of 12 to 19 mm on the day of trigger is used to more accurately assess trigger efficacy. Further prospective studies are required to identify whether administering the trigger by a measure other than lead follicle size can benefit outcomes, although such a benefit may only be apparent in patients with a wide distribution of follicle size during stimulation.

### Intrafollicular changes following hCG, GnRHa, or kisspeptin

Higher intrafollicular reproductive hormone levels have been associated with an improved chance of oocyte retrieval. Rosen *et al.* (173) observed that intrafollicular FSH levels were higher in follicles that yielded an oocyte. Lamb *et al.* (210) observed that oocytes fertilized by ICSI were 28% to 35% more likely to be retrieved from follicles with higher intrafollicular concentrations of estradiol and testosterone, whereas oocytes fertilized by IVF were 9% to 14% more likely to arise from follicles with higher estradiol or progesterone concentrations. Similarly, Itskovitz *et al.* (211) found that intrafollicular estradiol and progesterone levels were higher in follicles containing a mature oocyte. Interestingly, intrafollicular kisspeptin levels are higher than corresponding serum kisspeptin levels, and they correlate with follicular fluid estradiol levels and the number of mature oocytes retrieved (212). Haas *et al.* (213) assessed alterations in expression of genes related to steroidogenesis in granulosa cells of 24 women who received either GnRHa or hCG triggering. Expression of the enzymes CYP19A1 (0.50 vs 1) and CYP11A1 (0.6 vs 1), as well as 3*β*-hydroxysteroid-dehydrogenase (0.39 vs 1), vascular endothelial growth factor (VEGF; 0.74 vs 1), and inhibin *β* B (0.38 vs 1), was significantly lower in the GnRHa group (214). Expression of the FSH receptor was also significantly lower in the GnRHa group, but not expression of the LH receptor (213). Amphiregulin and epiregulin are ligands of the EGF receptor on mural granulosa cells, and amphiregulin’s expression was inversely related to fertilization rate (214). These EGF ligands have been proposed to be paracrine mediators of the LH signal to stimulate oocyte maturation (215). LH is known to stimulate upregulation of amphiregulin and epiregulin. Amphiregulin expression was 2.3-fold higher in mural granulosa cells in the GnRHa group, although not in follicular fluid (213). Expression of amphiregulin and epiregulin were both increased more than twofold in patients receiving both GnRHa and hCG in comparison with hCG alone (13). Expression of pigment epithelium-derived factor (an antiangiogenic factor secreted from granulosa cells) was also increased 1.5-fold, whereas cumulus cell conexin43 was reduced by 30% in the GnRHa-supplemented group (13).

Owens *et al.* (216) investigated expression of genes involved in ovarian reproductive function, steroidogenesis, and OHSS in granulosa lutein cells following the use of hCG, GnRHa, or kisspeptin to induce oocyte maturation in 48 women undergoing IVF treatment. Kisspeptin-54 increased expression of genes involved in ovarian steroidogenesis, the FSH receptor, the LHCG receptor, steroid acute regulatory protein (*STAR*), aromatase, estrogen receptors *α* and *β* (ESR1, ESR2), 3*β*-hydroxysteroid dehydrogenase type 2 (*3BHSD2*), and inhibin A, when compared with either hCG or GnRHa (216). Whereas *in vitro* treatment of granulosa lutein cells with hCG induced steroidogenic gene expression, kisspeptin-54 had no significant direct effects on either OHSS or steroidogenic genes (216).

*“The kisspeptin receptor has been hypothesized to play a key role in the pathogenesis of OHSS.”*

Although the increase in rates of OHSS with hCG have predominantly been ascribed to its longer duration of action, evidence for additional direct actions at the ovary may also be contributory. Neulen *et al.* (217) observed that hCG dose dependently induced VEGF expression in luteinized granulosa cells. Kitajima *et al.* (218) reported that GnRHa caused involution of corpora lutea of superovulated rats and reduced expression of VEGF, VEGF receptor 1, and VEGF receptor 2 and reduced vascular permeability in the ovaries of hCG-treated hyperstimulated rats. Similarly, hCG has been shown to directly increase VEGF expression and VEGF levels in human granulosa cells (217, 219, 220), whereas GnRHa may act directly on ovarian GnRH receptors to induce luteolysis (221, 222). Furthermore, the kisspeptin receptor has been hypothesized to play a key role in the pathogenesis of OHSS (223). Exogenous kisspeptin administration has been reported to reduce VEGF levels via a direct action on ovarian kisspeptin receptors to mitigate the risk of OHSS (223).

### Lessons from IVM

IVM is the process by which immature cumulus–oocyte complexes derived from antral follicles are matured *in vitro* (224). IVM was originally described in the context of unstimulated cycles without gonadotropin priming (225) and thus has been proposed as a useful option for women with PCOS who may have large numbers of small antral follicles putting them at increased risk of OHSS (226). There are typically three regimens used during IVM—the first is the original unstimulated cycle, whereby antral follicles are collected once follicles reach 10 to 12 mm in size before follicle dominance is established (227, 228). Alternative protocols, including priming with either FSH (229–231) or hCG (232, 233), have also been introduced in an attempt to increase oocyte yield and maturation rates, although controversy remains regarding whether these should be strictly thought of as IVM given that some *in vivo* maturation may also occur (224, 228).

However, IVM provides a unique opportunity to gain lessons on the size of follicle from which mature oocytes can be retrieved (228), as well as the optimal gonadotropin environment for oocyte maturation. Initial evidence from rodent studies suggested that meiosis was less likely to take place in oocytes retrieved from small follicles when cultured *in vitro* (234), with 83% to 91% of oocytes retrieved from antral follicles (300 to 600 μm in diameter) progressing to metaphase I or metaphase II, compared with only 2% of those from preantral follicles (100 to 150 μm in diameter) (234). However, in human studies, evidence of maturation potential has been observed in oocytes retrieved from follicles as small as 4 mm, and mature oocytes from follicles ≤10 mm following hCG priming had similar outcomes to those from larger follicles (235). Furthermore, evidence from studies priming follicles with hCG prior to retrieval has revealed that follicles <12 mm may possess granulosa cells with hCG receptors and can resume meiosis despite their small size (7). In their investigation of 238 hCG-primed IVM cycles in 213 patients with polycystic ovaries, Son *et al.* (235) reported that no significant difference in oocyte diameter, fertilization rate, or cleavage embryo quality was observed in oocytes obtained from follicles 10 to 14 mm, or those obtained from follicles <10 mm. Furthermore, 50.8% of oocytes retrieved from follicles <6 mm underwent oocyte maturation, with a fertilization rate of 63.7% (236).

Typically IVM has been undertaken in women at high risk of OHSS, although more recently, increased use for either fertility preservation in women undergoing cancer treatment or as a “rescue” treatment of women with poor ovarian reserve has been investigated. A prospective study compared 10 patients with normal ovarian reserve to 25 patients with poor ovarian reserve, and retrieved hCG-primed immature oocytes (237). For both normal responders, and those with low ovarian reserve, IVM increased the proportion of MII oocytes (237). At 24 hours, significantly greater proportion of germinal vesicles from women with low ovarian reserve had reached the MII stage, compared with those with normal ovarian reserve (237) (30.4% vs 66.9%; *P* = 0.013). However, fertilization rates and cleavage rates were similar between both groups.

In summary, the size of follicle from which mature oocytes can be retrieved can additionally be gleaned from studies of IVM (226). Follicles as small as 4 mm have been found to contain mature oocytes, and mature oocytes from follicles ≤10 mm following hCG priming resulted in similar outcomes to those retrieved from larger follicles (235). However, the rate of *in vivo–*matured oocytes positively correlates with dominant follicle size (dominant follicle ≤10 mm, 6.9%; 10 to 14 mm, 10.6%; >14 mm, 15.1%) (233). Similarly, Triwitayakorn *et al.* (238) observed that oocyte recovery rate increased from 57% of follicles <10 mm to 80% of follicles 10 to 14 mm and further to 86% of follicles >14 mm on the day of oocyte retrieval.

### Are gonadotropins mandatory for maturation?

It was originally demonstrated that human oocytes may persist at the germinal vesicle stage *in vitro* for up to 24 hours after collection, but that beyond this, they could resume meiosis independently of gonadotropins (227). However, given the variable maturation rates and cycle pregnancy rates in early IVM protocols (239, 240), the controlled addition of gonadotropins to culture medium was soon shown to improve the efficiency of IVM (239). Gonadotropins are hypothesized to exert their effect on oocyte maturation indirectly via follicular cumulus cells; however, oocytes possess gonadotropin receptors and thus may also act directly (241).

Studies have shown disparate results regarding the optimum ratio of FSH/LH required for IVM. Anderiesz *et al.* (242) found that the addition of recombinant FSH (rFSH) either alone or in combination with rLH in a ratio of 1:10 (to replicate gonadotropin concentrations during the endogenous mid-cycle LH surge) nonsignificantly increased oocyte maturation by 29% or by 39%, respectively (242). Choi *et al.* (243) found that cumulus expansion increased in proportion to concentrations of FSH and LH in a bovine animal model, and was maximal at 1 ng/mL FSH and 1 μg/mL LH. Hreinsson *et al.* (244) compared culture media supplemented with either 0.5 IU/mL hCG or 0.5 IU/mL LH and observed no significant difference in the proportion of oocytes that underwent maturation in the different cultures (55% hCG vs 56% LH). Although LH may not be a critical component of culture medium (245), activation of the LH receptor mediates cellular effects contributing to oocyte stability, with EGF a key mediator in transmitting LH receptor activation signaling to the cumulus cells and oocyte (246, 247). Another factor, brain-derived neurotropic factor expressed by granulosa cells following LH/hCG signaling, also increases oocyte maturation (248).

Several studies have suggested that kisspeptin may have additional direct effects at the ovary via ovarian kisspeptin receptors, beyond its predominant mode of action via endogenous GnRH release from the hypothalamus (163–165, 223). Castellano *et al.* (163) observed that kisspeptin expression increased in a cyclical manner during the menstrual cycle of a rodent model; kisspeptin was predominantly localized to the theca layer of growing follicles and the corpora lutea. Ovarian kisspeptin expression increased at ovulation, but was undetectable in immature oocytes (163). Kisspeptin has also been shown to increase IVM of ovine (164) and porcine immature oocytes and to increase blastocyst formation rate and blastocyst hatching (165). Although yet to be directly compared, oocyte maturation rates following kisspeptin appear comparable to other triggers despite serum LH levels achieved following kisspeptin seemingly being lower than those observed following rLH or GnRHa. Furthermore, there is a suggestion that kisspeptin may mature oocytes from smaller follicles than current triggers (166). However, kisspeptin has a short half-life, with circulating kisspeptin levels peaking at ~1 hour following subcutaneous injection and hence there is only a short duration of exposure to kisspeptin (18). Although one can speculate that kisspeptin could enhance oocyte maturation in combination with gonadotropin exposure through its predominant mode of action at the hypothalamus, it is unlikely that *in vivo* administration can lead to oocyte maturation in the absence of a gonadotropin response (16–18). Further studies investigating whether IVM of immature oocytes can be enhanced when kisspeptin is added to the culture medium would be of interest.

## Luteal Phase Characteristics Following Different Agents That Induce Oocyte Maturation

In the natural menstrual cycle, the luteal phase is defined as the period between ovulation and menstruation, or establishment of pregnancy (1). The corpus luteum secretes estrogen and progesterone to support the endometrium for implantation and placentation (9, 249). Stimulation of the LH receptor is required to maintain survival of the corpus luteum (250, 251), and inhibition of pituitary LH by either GnRHa (219, 251) or GnRH antagonist (251) results in luteolysis, with regression observed after 72 hours without LH activity (252, 253).

All IVF cycles are characterized by luteal phase dysfunction, and thus hormonal supplementation with luteal phase support, especially progesterone, is required to maintain adequate pregnancy rates (254, 255). hCG has a longer duration of action (14) and is able to better maintain survival of the corpora lutea than shorter acting triggers such as GnRHa (15, 35). Increased survival of corpora lutea following hCG improves endogenous sex steroid production and better maintains pregnancy rates (16, 18), but this comes at the expense of an increased risk of OHSS (220, 256). Despite GnRHa demonstrating a better safety profile, early studies with GnRHa were associated with reduced pregnancy rates and increased early pregnancy losses (108). For this reason, hCG is widely used as the preferred agent to induce oocyte maturation for most patients at low risk of OHSS, whereas GnRHa is predominantly reserved for patients at high risk of OHSS, although more intensive strategies to support the luteal phase are required.

### The luteal phase is deficient following all agents used to induce oocyte maturation

The luteal phase of all stimulated IVF cycles is dysfunctional (255, 257). When 40 women were randomized to receive either 250 μg of rhCG, 1 mg of rLH, or 0.2 mg of triptorelin to induce oocyte maturation without luteal phase supplementation, the luteal phase following all three was observed to be deficient (255). Although the oocyte maturation rate (proportion of oocytes that are mature) was comparable between the three groups (rhCG 85%, rLH 80%, and GnRHa 83%) (255), median serum LH on the day of oocyte retrieval significantly differed: rHCG 1.3 IU/L, rLH 50.6 IU/L, GnRHa 5.5 IU/L (255). The length of the luteal phase was best maintained by hCG: peak progesterone occurred on day 6 after rhCG, day 4 after rLH, and day 4 after GnRHa (*P* < 0.001), and the day of progesterone decrease was day 8 for rhCG, day 4 for rLH, and day 4 for GnRHa (255). The study was prematurely terminated due to low pregnancy rates (0% to 18%) in all three groups (255). Thus, even though the luteal phase is better preserved following hCG than other triggers, luteal phase supplementation is a mandatory component of all IVF cycles (255). The importance of progesterone for maintenance of pregnancy is long established, with early studies revealing that despite lutectomy, pregnancies could be supported by exogenous progesterone (258). Fanchin *et al.* (259) observed that increasing progesterone exposure was associated with reduced uterine contractility and increased pregnancy rates.

*“Some women could have a tendency toward a dysfunctional luteal phase regardless of the trigger used.”*

In 2004, Emperaire *et al.* (260) suggested that patients with a poor luteal phase in one ovulation induction cycle using GnRHa are likely to respond similarly in a subsequent cycle if GnRHa is used again. This remained the case even when the GnRHa dose was increased (0.5 mg) or given over three boluses of 0.1mg; however, luteal phase support with 1500 IU of hCG brought the luteal phase closer to normal (260). The authors therefore suggested that some women could have a tendency toward a dysfunctional luteal phase regardless of the trigger used (260).

### Routes of progesterone administration for luteal phase support

Vaginal progesterone results in lower circulating levels of progesterone when compared with intramuscular progesterone; however, local endometrial levels are much higher (261). As intramuscular progesterone can be uncomfortable, vaginal progesterone is more often used, especially in hCG-triggered patients (262). As progesterone produced by the ovary normally reaches the endometrium via the peripheral circulation, parenteral progesterone has been suggested as being more similar to physiological pathways. Oral progesterone was initially suggested as luteal phase support during the 1980s, but early studies demonstrated a lack of endometrial secretory changes when compared with those receiving intramuscular or vaginal progesterone due to significant first-pass metabolism (263, 264). Recently, dydrogesterone, a metabolite of progesterone possessing biological activity and good oral bioavailability, has been shown to have similar efficacy to vaginal progesterone in hCG-triggered patients (265).

### Luteal phase support following different agents of oocyte maturation

In 2005, Humaidan *et al.* (24) randomized 122 women to receive either GnRHa or hCG to induce oocyte maturation. All received luteal phase support in the form of vaginal progesterone at 90 mg daily, and estradiol at 4 mg daily orally (24). Mean serum hormonal levels at 7 days after oocyte retrieval for GnRHa- and hCG-treated patients were as follows: LH (1.5 IU/L vs 0.2 IU/L), FSH (1.9 IU/L vs 0.4 IU/L), estradiol (2.9 nmol/L vs 7.1 nmol/L), and progesterone (39 nmol/L vs 283 nmol/L) (108). Clinical pregnancy rates per cycle were significantly reduced following GnRHa compared with hCG (6% vs 36%) (24). Thus, there was a recognition that the luteal phase required more intensive support following GnRHa-triggered cycles than hCG-triggered cycles.

One approach pioneered by Engmann *et al.* (266) was to use high-dose sex steroids with intensive intramuscular progesterone and estradiol supplementation. In an RCT comparing GnRHa and hCG to induce oocyte maturation in 66 women at high risk of OHSS, luteal phase support was provided intramuscularly by 50 mg of progesterone titrated up to 75 mg intramuscularly to maintain serum progesterone levels > 20 ng/mL (63.6 nmol/l) and 3 × 0.1-mg transdermal estrogen patches on alternate days titrated up to four patches and 2 mg of oral estrogen twice daily to maintain serum estradiol level >200 pg/mL (734.2 pmol/L) (266). Serum progesterone and estradiol levels were both lower on the day of embryo transfer following GnRHa (serum estradiol, 485 pg/mL vs 1320 pg/mL; serum progesterone, 25 ng/mL vs 117 ng/mL) (266). However, the implantation rate (36% vs 31%), clinical pregnancy rate (56.7% vs 51.7%), and ongoing pregnancy rate (53.3% vs 48.3%) per transfer following GnRHa and hCG were similar (266). Unfortunately, other investigators were not able to replicate the same excellent pregnancy rates with this intensive luteal phase support regimen (267, 268). A retrospective cohort study compared 257 women at high risk of OHSS (≥15 follicles ≥12 mm on day of trigger) triggered with hCG and 363 women triggered with GnRHa triptorelin at 0.2 mg with intensive luteal phase support (intramuscular progesterone at 50 mg daily and vaginal progesterone at 90 mg twice daily and 6 mg of estradiol valerate) (269). Live-birth rates were similar (GnRHa 29.8% vs hCG 29.2%) between the groups, but although one late-onset severe OHSS case was observed in the GnRHa group (0.3%), 18 (7%) were observed after hCG (269). If luteal phase support strategies can be shown to reliably maintain pregnancy rates following GnRHa to the same extent as hCG, then the preferable safety profile of GnRHa could encourage its use as a first-line agent more widely.

### Use of hCG for luteal phase support

Given the reduction in pregnancy rates with GnRHa, there is great interest in supporting the luteal phase using a small dose of hCG either given at the same time as GnRHa or at an interval during the early luteal phase to stimulate endogenous progesterone production.

Four oocyte donors underwent four oocyte donor cycles within 1 year to assess the luteal phase characteristics following different regimens (270). Following the short protocol, women received one of the following regimens: (1) hCG at 10,000 IU to induce oocyte maturation, followed by standard LPS (600 mg of vaginal progesterone three times daily and 4 mg of estradiol valerate daily from the day after oocyte retrieval), (2) GnRHa (0.2 mg of triptorelin) and 1500 IU of hCG 35 hours thereafter with standard LPS, (3) GnRHa (0.2 mg of triptorelin), or (4) GnRHa without luteal phase support (270). Estradiol and progesterone levels were higher at 5 days following oocyte retrieval in women who either received hCG either to induce oocyte maturation or as part of LPS (270). Estradiol on day 5 following oocyte retrieval was 1862 ng/L (10,000 IU of hCG plus LPS), 1238 ng/L (GnRHa at 0.2 mg of triptorelin plus 1500 IU of hCG), 132 ng/L (GnRHa plus LPS), and 66 ng/L (GnRHa) (270). Progesterone on day 5 following oocyte retrieval was also lower at 60 μg/L (10,000 IU hCG plus LPS), 60 μg/L (GnRHa at 0.2 mg of triptorelin plus 1500 IU of hCG), 11.49 μg/L (GnRHa plus LPS), and 0.99 μg/L (GnRHa) (270). Thus, a small dose of hCG given 35 hours following GnRHa was able to simulate the luteal phase characteristics of hCG-triggered cycles.

In 2010, Humaidan *et al.* (271) reported that administration of 1500 IU of hCG at the time of oocyte retrieval was sufficient to support the luteal phase in those receiving GnRHa to a similar extent as those receiving 10,000 IU of hCG to induce oocyte maturation. In 2013, Humaidan *et al.* (272) randomized women at high risk of OHSS (15 to 25 follicles ≥11mm) to receive either (1) GnRHa buserelin at 0.5 mg followed by a single bolus of 1500 IU of hCG for luteal phase support (n = 60), or (2) to 5000 IU of hCG (n = 58). Women assessed as not being at high risk of OHSS (<15 follicles ≥11 mm) were randomized to receive either (3) GnRHa buserelin at 0.5 mg followed by hCG at 1500 IU on the day of oocyte retrieval and a further dose 5 days thereafter (n = 125), or (4) hCG at 5000 IU (n = 141) (272). All women also received micronized progesterone at 90 mg twice daily (272). There was no significant difference in pregnancy rates between groups (ongoing pregnancy rate per randomization of 25% to 30%) (272). Two cases of moderately late OHSS occurred in both group 2, who received hCG, and a further two cases in group 3, who received two small doses of hCG for luteal phase support (272). However, Seyhan *et al.* (273) reported high rates of severe OHSS (6 of 23) when hCG supplementation was used in patients at increased risk of OHSS, and thus this approach is not recommended in patients with a very large number of follicles on the day of trigger (23).

Kol *et al.* (274) investigated whether luteal phase support could be provided by hCG supplementation alone in the absence of progesterone supplementation. Fifteen patients were triggered with GnRHa (triptorelin at 0.2 mg) and received 1500 IU of hCG following oocyte retrieval and again 4 days later, achieving an ongoing clinical pregnancy rate of 47. Similarly, Andersen *et al.* (275) conducted a proof-of-concept study in 93 women, demonstrating that low-dose hCG (150 to 200 IU daily) could be used to generate endogenous progesterone production and support pregnancy without the need for exogenous progesterone.

Thus, although the luteal phase may be insufficient following GnRHa trigger in many patients, this may not be universal. A recent study sought to investigate this concept, termed “luteal coasting,” whereby the luteal phase is monitored and a rescue dose of hCG is administered only when progesterone levels drop (276). Three women at high risk of OHSS received a short protocol with 0.3 mg of triptorelin to induce oocyte maturation (276). Serum progesterone was measured 48 hours after oocyte retrieval, and supplemental hCG was administered at varying doses when serum progesterone dropped to <15 ng/mL (282). In two out of three patients, this approach was sufficient to support the luteal phase (276). In a further observational study by Lawrenz *et al.* (277), 51 women at risk for OHSS received GnRHa (0.3 mg of triptorelin) to induce final oocyte maturation, and vaginal progesterone supplementation was started from the night of oocyte retrieval and continued at 400 mg three times daily thereafter. Serum progesterone was measured 48 hours after oocyte retrieval and used to assess whether participants required additional luteal support in the form of hCG supplementation (277). Mean serum progesterone 48 hours after administration of GnRHa was 33.43 ng/mL (range, 13 to 60 ng/mL) (277). Thus, luteal phase deficit can be variable, and in the future tailored supplementation regimens may be developed (277).

### Use of GnRH agonist for luteal phase support

GnRH receptors are present throughout the endometrium in stromal and epithelial cells, and their expression is increased during the secretory phase (278, 279), as are LH receptors (280, 281). Hypotheses for the reduced pregnancy rates observed following GnRHa have included those related to factors aside from the duration of the LH surge, such as a direct endometrial action to prevent implantation, or a direct induction of luteolysis via GnRH receptors. However, there is evidence to suggest that GnRHa can be used as luteal phase support. A prospective placebo-controlled study by Tesarik *et al.* (282) investigated the effects of GnRHa administration at the time of embryo transfer in oocyte donor cycles, using either 0.1 mg of triptorelin 3 days following embryo transfer or placebo. The implantation rate was significantly higher (36.9% vs 25.1%, *P* < 0.05) in women receiving GnRHa on the day of embryo transfer (282). Interestingly, intravenous administration of GnRH at 100 μg during pregnancy can stimulate production of hCG from the placenta (289). In 2006, Pirard *et al.* (281) found that similar numbers of patients achieved clinical pregnancy following hCG at 10,000 IU with 200 mg of micronized progesterone compared with those receiving buserelin at 200 μg and then 100 μg three times daily (two of five vs three of five, respectively). Despite the small sample size, this study suggested that low-dose GnRHa could be used to support the luteal phase (281).

*“Improving luteal phase support regimens following GnRHa to achieve reliable pregnancy rates can extend the use of these agents.”*

A randomized prospective study investigated the effect of mid-luteal administration of GnRHa in both 300 short and 300 long protocols, where hCG had been used to induce oocyte maturation (282). Women were randomized to receive either GnRHa or placebo 6 days after oocyte retrieval (282). All women received 4 mg of estradiol daily and 400 mg of vaginal micronized progesterone daily from the day of oocyte retrieval for 17 days, and additionally received 250 μg of rhCG on the day of embryo transfer (282). Both estradiol and progesterone levels were greater at 7 days following oocyte retrieval in the GnRHa-treated patients (estradiol, GnRHa at 405 pg/mL, placebo at 372 pg/mL; progesterone, GnRHa at 42 ng/mL, placebo at 29 ng/mL) (282). Implantation rates were 29.8% luteal phase GnRHa vs 18.2% placebo (*P* < 0.05) and live birth rates per intention to treat were 27.4% luteal phase GnRHa vs 18.2% placebo (*P* < 0.05) (282). However, to date the use of GnRHa for luteal phase support is not widely used in practice.

In summary, all agents of oocyte maturation can induce luteal phase defect and require luteal phase support (255). However, luteal phase deficit is more pronounced following GnRHa than hCG (24), and thus more intensive luteal phase support is required. Intensive luteal phase support with high-dose sex steroid supplementation is an attractive option, as this strategy will not increase the risk of OHSS (266). Conversely, care must be taken when using even a small dose of hCG for luteal phase support in women at very increased risk of OHSS to maintain the benefit for safety in avoiding hCG triggering (273). Improving luteal phase support regimens following GnRHa to achieve reliable pregnancy rates can extend the use of these agents more widely in place of hCG.

## OHSS Following Different Agents That Induce Oocyte Maturation

OHSS is one of the most common complications of IVF treatment (285) and is predominantly related to the use of hCG to induce final oocyte maturation (11). The prolonged duration of action of hCG results in overstimulation of the ovaries and the release of vasoactive substances from the ovary, particularly VEGF-A, which causes leakage of fluid from the vascular space into the third spaces of the body (11). Thus, OHSS is a potentially life-threatening iatrogenic condition that can result in massive ovarian enlargement, ascites, hydrothorax, renal failure, acute respiratory distress syndrome, and rarely even death (estimated at 3 per 100,000) (286).

The most commonly used diagnostic criteria for OHSS are those of Golan *et al.* (287) from 1989 with the updated categorization by Navot *et al.* (288) in 1992. Mild OHSS is reported to occur in one third of cycles, moderate OHSS in a tenth, and severe OHSS in 2% of IVF cycles using hCG to induce oocyte maturation (11). Mild OHSS predominantly consists of symptoms alone and is likely to resolve with conservative management. Hence, mild OHSS is not regarded as clinically significant by some practitioners and is often not reported (11). Moderate OHSS is characterized by the additional presence of ascites on ultrasound, and severe OHSS as additionally having evidence of hemoconcentration, renal impairment, or respiratory distress (11).

A further subcategorization of OHSS is used to reflect a difference in pathophysiology by the time of onset following oocyte retrieval: “early OHSS’ occurs within 9 days of oocyte retrieval, whereas “late OHSS” occurs 10 days or more following oocyte retrieval (289–291). Early OHSS relates to the use of hCG to induce final oocyte maturation (or for luteal phase support), whereas late OHSS relates to endogenous hCG production from a developing pregnancy and thus can be further exacerbated by multiple pregnancy (289). Consequently, early OHSS can be prevented through the use of alternate triggers of oocyte maturation than hCG (12, 16), whereas late OHSS can be mitigated by segmentation (cryopreservation of all embryos with embryo transfer in a subsequent cycle) and avoidance of multiple transfers (292). However, even the use of GnRHa for inducing oocyte maturation and in combination with segmentation does not completely eliminate the risk of severe OHSS (293–299).

Late OHSS is often more severe and harder to manage than early OHSS as the stimulus for hCG production is ongoing (pregnancy). Whereas late OHSS is often considered a separate entity to early OHSS, it is noteworthy that late OHSS almost never occurs in the context of frozen embryo transfers where ovarian stimulation has not recently been carried out, even in high-risk patients, suggesting that late OHSS represents an exacerbation of subclinical early OHSS by subsequent pregnancy-related hCG production. Consequently, the use of alternative triggers to hCG can be expected to reduce the risk of late OHSS as well as early OHSS. Increased use of segmentation can reduce the occurrence of late OHSS; however, the “risk of OHSS” remains one of the most frequent reasons for cycle cancellation prior to embryo transfer across the world. In Europe, 7.1% of the 387,399 IVF cycles started in 2012 were cancelled prior to oocyte retrieval and 19.4% of cycles commenced did not have a fresh embryo transfer (300). Similarly in the United States, 10.5% of 92,862 IVF cycles commenced in 2014 were cancelled prior to oocyte retrieval, of which 5% were due to “ovarian overresponse” and 27.8% of IVF cycles were segmented (301). In the United Kingdom, >5% of cycles were cancelled prior to oocyte retrieval due to risk of OHSS, and this was also the most common reason for cycle cancellation between oocyte retrieval and embryo transfer (40% of cycle cancellations at this stage) (302).

Rates of OHSS from retrospective studies relying on patient-initiated presentation for assessment could lead to underreporting of OHSS rates in comparison with studies where routine assessments are made (41). In a well-conducted prospective clinical trial, severe OHSS occurred in 5.1% to 8.9% of patients and moderate OHSS in a further 10.2% to 15.6% of patients depending on whether a short or long protocol was used (41). Despite these high rates of clinically significant OHSS (up to 24.5%), these were rates of OHSS in an unselected population not at increased risk of OHSS (41). In the 11% of patients with “irregular cycles” (implying the presence of polycystic ovarian syndrome), the rate of severe OHSS was further increased to 13.9% (41).

In 2005, Shapiro *et al.* (303) retrospectively analyzed 849 IVF cycles in which an hCG dose between 2500 IU and 20,000 IU was used depending on each patient’s weight and OHSS risk to evaluate whether hCG level could predict OHSS risk. Serum hCG levels ranged between 55 and 530 IU/L at 12 to 16 hours following hCG (303). Of 849 cycles, 27 were diagnosed as OHSS (3.2%) and 12 required paracentesis (1.4%) (303). Patients with OHSS had a mean serum hCG of 172 IU/L (range, 37 to 731 IU/L) (303). The number of follicles on the day of hCG and the serum hCG levels were independent predictors of OHSS (303). The risk of OHSS in patients with 45 follicles on the day of hCG increased from 50% in those with serum hCG at 100 IU/L, to 62% when serum hCG was 200 IU/L, to 72% when serum hCG was 300 IU/L, and to 80% when serum hCG was 400 IU/L (303). The risk of OHSS in patients with 35 follicles on the day of hCG increased from 10% in those with serum hCG at 100 IU/L, to 17% when serum hCG was 200 IU/L, to 25% when serum hCG was 300 IU/L, and to 36% when serum hCG was 400 IU/L (303). Patients with 25 follicles had an increase in risk from 2% to 6% with hCG level, whereas patients with 15 follicles on the day of hCG had minimally increased risk even with higher serum hCG levels (303). Thus, both serum hCG level as well as number of follicles influenced the risk of OHSS.

Fábregues *et al.* (304) compared the characteristics of IVF cycles of 22 women who were diagnosed with severe OHSS in their first long protocol IVF cycle, but not in a subsequent cycle within 12 months. During 10 years the incidence of OHSS at the center was 1.5% (62 of 4065 cycles) and 41% of patients included in this study had PCOS (304). Patients received intramuscular hCG 35 to 36 hours prior to oocyte retrieval, coasting up to 4 days and transfer of up to 4 embryos (304). Patients had more mature oocytes retrieved in the OHSS cycle (15.1 vs 8.0) and higher estradiol levels on day of hCG (4242 vs 2459 pg/mL), but they had similar implantation rates (12% to 15%) (304). This study suggests that careful management of cycles in women at risk for OHSS can help to mitigate the risk in subsequent cycles (304).

In 2000, Mathur *et al.* (291) conducted a retrospective analysis of rates of OHSS in 2362 cycles in 1565 patients with serum estradiol levels <15,000 pmol/L and <30 follicles ≥12 mm in diameter who received 5000 IU of hCG 36 hours prior to oocyte retrieval. In patients with <19 oocytes retrieved or serum estradiol <10,000 pmol/L, hCG at 2500 IU was administered on the day of embryo transfer (291). Early OHSS occurred in 48 cycles (2%) and late OHSS in a further 1.3% at a median time of 7 days after oocyte retrieval (291). Patients with OHSS had more oocytes retrieved (median 13 vs 9) and were more likely to have PCOS (12.8% vs 4.1%) (291). The incidence of early OHSS increased with the number of oocytes retrieved from ~1% in those with 5 to 9 oocytes to ~7% in those with >19 oocytes (291). Late OHSS rates also rose, but to a lesser extent (~3% in those with >19 oocytes) (291). The number of oocytes predicting early moderate to severe OHSS was nine (positive likelihood ratio 1.95, negative likelihood ratio 0.21) (291). Serum estradiol level on day of hCG predicting early moderate to severe OHSS was 6782 pmol/L (sensitivity 91%, specificity 69%, positive likelihood ratio 2.88, negative likelihood ratio 0.13) (291). These and similar data help to inform the risk of OHSS following an hCG trigger. Although the number of follicles on day of trigger is the best predictor of subsequent OHSS, the cutoffs are not absolute and there remains uncertainty in the subsequent risk of OHSS. Other markers of increased risk of OHSS include serum AMH, AFC, estradiol levels, number of intermediately sized follicles on day of trigger, and number of oocytes retrieved (290, 305–308).

### OHSS risk in high-risk populations

Women with polycystic ovaries have an approximately fivefold increase in risk of OHSS (309). MacDougall *et al.* (310) observed that polycystic ovaries on ultrasound were found in 63% of severe OHSS cases and in 57% of moderate OHSS cases compared with 33% of the general patient population. The use of GnRHa to induce final oocyte maturation can significantly reduce the incidence of OHSS in comparison with hCG; however, a number of case reports have suggested that severe OHSS may still occur in the high-risk patient even when triggered with a GnRHa and treated with segmentation (293–299). A retrospective analysis of SART database by Steward *et al.* (306) in 2014 reported that retrieval of at least 15 oocytes was predictive of OHSS risk. Swanton *et al.* (311) reported that patients with PCO morphology or PCOS had between 14 and 16 oocytes retrieved, and reported severe OHSS rates of 12.6% to 15.4%. Similarly, Jacob *et al.* (312) recently reported a clinical trial in women with PCOS; despite a median of 14 to 15 oocytes being retrieved, the study reported moderate/severe OHSS rates of 12% to 16%, which the authors state may have been an underestimate due to a lack of routine screening (312). Furthermore, 12 of 153 patients were cancelled due to risk of overresponse (312).

*“GnRHa is preferable to hCG in the patient at increased risk of OHSS.”*

In 2016, Krishna *et al.* (313) conducted a randomized unblinded study of 227 women under the age of 37 years who met the Rotterdam criteria for PCOS. Patients with serum E2 <6000 pg/mL received either GnRHa at 0.2 mg of triptorelin (n = 92) or 250 μg of hCG (n = 101) to induce oocyte maturation (313). Approximately 50% of patients were oligomenorrheic, mean AFC was 25 to 26, mean AMH was 5.7 ng/mL in the GnRHa group, and 4.4 ng/mL in the hCG group (313). There were 24.4 follicles >14 mm in the GnRHa group and 19.8 follicles >14 mm in the hCG group (313). Only 1 patient (1%) was diagnosed with mild OHSS in the GnRHa group whereas in the hCG group, only 10 patients (9.9%) were not diagnosed with OHSS: 52% had mild OHSS, 35% had moderate OHSS, and 3% had severe OHSS (313). Patients in the GnRHa group had more oocytes retrieved (23.5 vs 20.8), more mature oocytes (19.1 vs 14.1), a higher oocyte maturation rate (82% vs 73%), and a higher proportion of patients had a top quality cleavage embryo formed (91% vs 74%) (313). A calculated mature oocyte yield from aggregated data (number of mature oocytes divided by number of follicles >14 mm) was 78% in the GnRHa group and 71.2% in the hCG group (313). Thus, GnRHa is preferable to hCG in the patient at increased risk of OHSS.

To date there have been two clinical trials investigating the use of kisspeptin in populations at high risk of OHSS comprising 122 patients (16, 17). Women <35 years old and BMI <30 kg/m^2^ were identified as being high risk for OHSS by serum AMH level ≥40 pmol/L or AFC ≥23 and received a single subcutaneous bolus of kisspeptin-54 at doses of 3.2 to 12.8 nmol/kg (16, 17). In the first trial of 60 women at increased risk of OHSS, 75% of patients had an AMH ≥40 pmol/L, all patients had an AFC ≥23, and 42% had an AFC ≥40 (26). Furthermore, 88% of women had >14 follicles and 28% of women had >25 follicles (26). A quarter of the women (n = 15) had previously had an IVF cycle using hCG to induce oocyte maturation, and 20% (3 of 15) of them had developed severe OHSS requiring admission to a hospital for medical intervention or intensive care support (26). Despite the high risk of the cohort, only 5% were diagnosed with mild early OHSS and 2% with mild late OHSS, but no patient was diagnosed with moderate to severe OHSS (26). The second trial using kisspeptin in a cohort of women at high risk of OHSS included 62 women with the same inclusion criteria (26). Women received either one or two doses of kisspeptin 10 hours apart (26). Despite a second dose of kisspeptin extending the LH surge, there was no increase in the rates of OHSS (26). One woman was diagnosed with moderate early OHSS in single group (1.6%), and one mild late OHSS (1.6%) in the double group (26).

A single center retrospective cohort study compared clinical parameters of OHSS in hCG (n = 40), GnRHa (n = 99), or kisspeptin-54 (n = 122) in women at risk for OHSS identified by AFC >23 or total number of follicles on day of trigger >23 (314). Women had a median of 38 antral follicles, 24 follicles ≥11 mm on the day of trigger, and 19 oocytes retrieved (314). Median ovarian volume at 3 to 5 days after oocyte retrieval was larger following hCG (138 mL) than GnRHa (73 mL; *P* < 0.0001), and in turn kisspeptin (44 mL; *P* < 0.0001) (314). Median ovarian volume remained enlarged 20-fold following hCG, 8-fold following GnRHa, and 5-fold following kisspeptin compared with prestimulation ovarian volumes (314). Mean (±SD) ascitic volumes were lesser following GnRHa (9 ± 44 mL) and kisspeptin (5 ± 8 mL) than hCG (62 ± 84 mL; *P* < 0.0001) (314). Symptoms were most frequent following hCG and least frequent following kisspeptin (314). Moderate to severe OHSS occurred in 37.5% of patients following hCG, 3% following GnRHa, and no patient following kisspeptin (314). The OR for OHSS was 33.6 (CI, 12.6 to 89.5) following hCG and 3.6 (CI, 1.8 to 7.1)following GnRHa, when compared with kisspeptin (314). These data are consistent with a proposed role for kisspeptin in the pathogenesis of OHSS beyond that due to duration of action. Exogenous kisspeptin administration has been reported to reduce VEGF levels via a direct action on ovarian kisspeptin receptors to mitigate the risk of OHSS (225). Nevertheless, the reduced rates of OHSS following kisspeptin observed during the trials so far require verification in prospective studies directly comparing kisspeptin to current triggers of oocyte maturation. Kisspeptin analogs are currently in development and may allow for a further novel triggering option in the future.

## Summary

The mode by which oocyte maturation is induced has a significant impact on the ability to retrieve mature oocytes, the luteal phase characteristics predicating implantation, and the risk of OHSS. An appreciation of the endocrine and temporal requirements for oocyte maturation enables the optimization of current IVF protocols and the development of novel approaches to induce oocyte maturation to improve both the safety and efficacy of IVF treatment.

## Abbreviations

AFC: antral follicle count
AMH: anti-Müllerian hormone
BMI: body mass index
CDK1: cyclin-dependent kinase 1
cGMP: cyclic guanosine monophosphate
EFS: empty follicle syndrome
EGF: epidermal growth factor
GnRHa: GnRH agonist
GnSAF: gonadotropin surge-attenuating factor
GVBD: germinal vesicle breakdown
hCG: human chorionic gonadotropin
ICSI: intracytoplasmic sperm injection
IQR: interquartile range
IVF: *in vitro* fertilization
IVM: *in vitro* maturation
OHSS: ovarian hyperstimulation syndrome
RCT: randomized controlled trial
rFSH: recombinant FSH
rhCG: recombinant human chorionic gonadotropin
rLH: recombinant LH
*t*_1/2_: half-life
uhCG: urinary human chorionic gonadotropin
VEGF: vascular endothelial growth factor
